# Main-group metal cyclophane complexes with high coordination numbers†

**DOI:** 10.1039/d0ra05303a

**Published:** 2020-08-20

**Authors:** Yasir Altaf, Muhammad Yar, Muhammad Ali Hashmi

**Affiliations:** School of Chemical and Physical Sciences, Victoria University of Wellington New Zealand; Department of Chemistry, COMSATS University Islamabad, Abbottabad Campus KPK 22060 Pakistan; Department of Chemistry, University of Education, Attock Campus Attock 43600 Pakistan muhammad.hashmi@ue.edu.pk

## Abstract

Density functional theory calculations using the PBE0-D3BJ hybrid functional have been employed to investigate the complexation of main-group metal-cations with [2.2.2]*para*cyclophane and deltaphane. Geometry optimization under symmetry constraints was performed to observe the mode of coordination that a metal-cation adopts when it resides inside the cyclophane cavity. Thermodynamic properties were investigated to note the trends of stability along a group of metals. To further investigate the bonding properties, Morokuma–Ziegler energy decomposition analysis, natural bond orbital analysis and Bader's analysis were employed. It was observed that most of the main-group metal complexes with cyclophanes prefer an η^6^η^6^η^6^ coordination mode where the metal-cation sits in the centre of the cyclophane cavity. There is an increased thermodynamic stability in [2.2.2]*para*cyclophane complexes compared to their deltaphane analogues while the reverse is true regarding the strength of coordination based on interaction energy.

## Abstract 2

Investigation of the complexation of main-group metal-cations with [2.2.2]*para*cyclophane and deltaphane.

## Graphical abstract

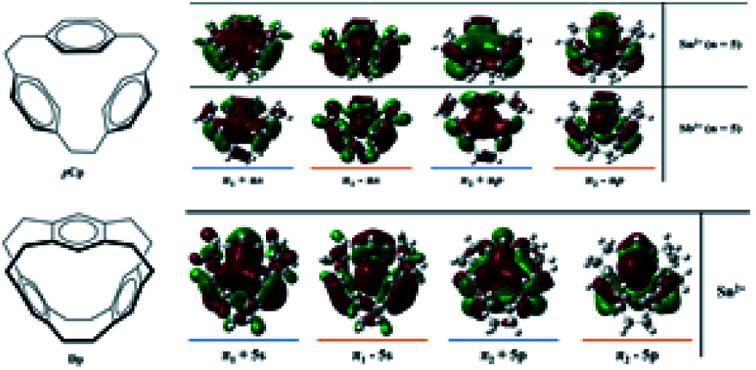

## Introduction

Cyclophanes consist of two or more aromatic rings connected through aliphatic bridges, to form a cyclic cavity.^1^ The aromatic rings are characterized by a planar geometry but the strain imposed by the aliphatic components renders the geometry of the cyclophanes twisted.^2^

There is a growing interest in the donor–acceptor complexes of cyclophanes with metals due to their various applications such as the development of ion-selective electrodes,^3^ catalysis^4^ and chelation.^5^ Metal-chelating agents involving cyclophanes are potential candidates to be used in waste-water treatment since they are known for metal-ion scavenging.^3,6^ Host molecules undergo conformational changes upon incorporation of the guest species in their macrocyclic cavity and these differences may be observed sometimes through fluorescence. This allows recognition of targetted guest metal-cations.^7^ Moreover, derivatives of metallacyclophane hosts have been reported as potential candidates for optical biomolecular recognition.^8^ Luminescent metal-complexes of cyclophanes have been reported to have their potential applications in thin-film nonoporous materials.^9^ All these applications are due to the potential of the π-rich cyclophane cavity to host electrophilic or cationic guests by making inclusion complexes, despite the fact that some smaller cyclophanes are also known to form exclusion complexes.^10^

**Fig. 1:**
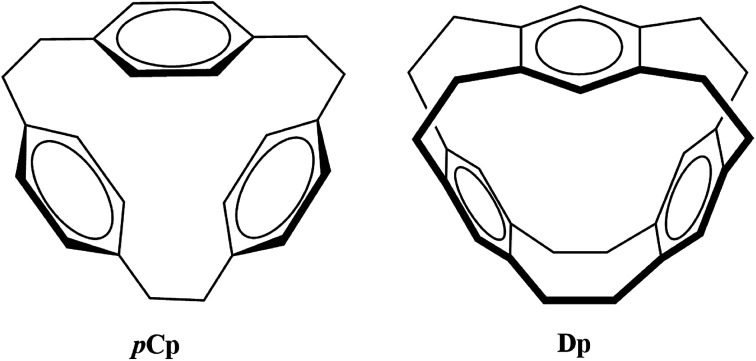
Cyclophane ligands of interest in the current study.

The selectivity and sensitivity of cyclophanes can be improved as evident from the efforts in the past such as derivatization and functionalization using different coordination groups,^4,11,12^ controlling the size of the cavity to capture guest species^13^ and replacing the usual phenyl ring with heterocyclic aromatic rings such as imidazolium.^14^ Owing to the variety of their potential applications, it is important to explore the bonding properties of different possible metallacyclophanes. In the current study, the two cyclophanes [2.2.2]*para*cyclophane (*p*Cp) and deltaphane (Dp) given in Fig. 1 were selected for this purpose.

*p*Cp was first synthesized by Pierre and co-workers where they called it a π-prismand due to its π-rich prism-shaped cavity^15^ and demonstrated its complexation with silver triflate. Extending the concept and introducing an increased rigidity compared to that of *p*Cp, Kang *et al.* reported the synthesis of Dp.^16^ As the systematic name of the latter *i.e.* [2,2,2,2,2,2](1,2,4,5)cyclophane suggests, Dp has six ethano bridges compared to three in *p*Cp. *p*Cp and Dp consist of three phenyl rings each bridged through aliphatic chains. However, *p*Cp is more flexible as each aromatic ring is attached to two ethyl bridges unlike its attachment to four in case of Dp.

Due to the presence of phenyl rings, both these molecules contain π-rich cavities that can coordinate to the metal-cations to form stable complexes. It was shown earlier that the silver triflate complex of *p*Cp is much more stable compared to other such complexes with aromatic systems.^15^ Similarly, the crystal structure of silver triflate complex of Dp was also reported.^16^ During the same period (mid to late 1980s), Schmidbaur and co-workers reported the groundbreaking complexes of *p*Cp with some of the main-group metals.^17–19^ Despite these contributions in synthesis of metallacyclophanes of *p*Cp and Dp, a lot of effort is still required to explore the bonding properties of these complexes to make use of these promising π-donating ligands on industrial scale.

In this regard, we previously reported the bonding properties of coinage metal complexes of *p*Cp and Dp.^20^ Earlier, the groups of Frenking and Castro carried out a computational study on the coordination mode and bonding properties of inclusion complexes of Sn^2+^ and Ag^+^ with *p*Cp.^21^ They further extended the concept to computationally understand the role of formal charge of a cation in π–cation interactions by comparing the complexes of isoelectronic In^+^ and Cd^2+^ with *p*Cp.^22^ In further instances, Castro *et al.* investigated helicenes^23^ and Dp^24^ as potential π-donors to form various π–cation interactions through relativistic DFT approach. A review of the use of relativistic computational tools to study the structural and bonding properties of these π–cation interactions has been published recently^25^ which shows that different avenues in this field have been opened by the groups of Castro and Frenking over the last few years. The current work is aimed at investigating the coordination and bonding properties of the complexes of group 13 (Ga^+^, In^+^ and Tl^+^), 14 (Ge^2+^, Sn^2+^ and Pb^2+^) and 15 (As^3+^, Sb^3+^ and Bi^3+^) with the π-rich cavity of *p*Cp and Dp using density functional theory (DFT).

### Computational details

All calculations related to geometry optimization were performed with the *Gaussian 09* suite of programs (Revision D.01).^26^ The PBE0 hybrid functional^27^ in conjunction with Grimme's empirical D3 correction with Becke–Johnston damping (D3BJ)^28^ was used in combination with the def2-TZVP basis-set^29^ of triple-*ζ* quality in all these calculations.

The optimized structures were further subject to the Morokuma–Ziegler Energy Decomposition Analysis (MZEDA)^30^ that was carried out with the ADF2014 program.^31^ The TZ2P (Slater Type Orbital) basis set^32^ was employed along with the relativistic ZORA Hamiltonian.^33,34^ MZEDA involves the decomposition of total energy (Δ*E*) of a molecule as:

1 Δ*E* = (Δ*E*_1_ + Δ*E*_2_) + Δ*E*^int^

In eqn (1), Δ*E*^int^ is the instantaneous interaction between the two molecular fragments and (Δ*E*_1_ +Δ*E*_2_) is the sum of their individual energies. The above equation implies that Δ*E*^int^ is the difference between the total energy of a molecule and its fragments. Δ*E*^int^ can be further subdivided as in eqn (2):

2 Δ*E*^int^ = Δ*E*^Pauli^ + Δ*E*^elstat^ + Δ*E*^orb^

Here, Δ*E*^elstat^ is the energy due to electrostatic interactions (mostly attractive in nature) between the molecular fragments. Δ*E*^Pauli^ is the repulsion term and it arises due to the electrons with same spin. Δ*E*^orb^ indicates the interactions involving charge transfer polarization effects.

Bader's analysis based on quantum theory of atoms in molecules (QTAIM)^35^ given by Richard Bader was performed using Multiwfn software.^36^ NBO analysis was carried out with *NBO 6.0* program^37^ as interfaced with *Gaussian09*.

Molecular graphics were rendered with GaussView 5.0.9.^38^

## Results and discussion

### Structural features

#### Un-complexed cyclophanes

*p*Cp possesses a rigid geometry and has an internal cavity with diameter 2.5 Å.^39^ The phenyl rings are bridged at the *para* positions through ethyl chains. Dp is even more rigid as the phenyl rings are doubly connected to each other at the *ortho* and *meta* positions through ethano bridges. The *D*_3_ symmetric geometries were optimized at PBE0-def2TZVP level of DFT and have been characterized as minima on the potential energy surface. Additionally, the two have *D*_3h_ symmetric transition structures optimized previously at the same level.^20^ Calculated structural data of both of these ligands is consistent with their crystal structural information^16,39^ except the fact that gas-phase calculated bond lengths are sometimes longer than those in a crystal structure (solid-phase) due to crystal packing forces that are absent in a gas-phase calculation.

In *p*Cp, the C–C bond lengths in bridging ethyl units and that for bridgeheads are 1.54 Å and 1.51 Å compared to the experimental mean bond lengths of 1.43 Å and 1.52 Å respectively. The calculated C–C bond lengths in the phenyl rings are equivalent at 1.39 Å compared to the experimental bond lengths *i.e.* 1.37 Å. Dp presents a different case with a small variation of bond lengths in phenyl rings. Here, the calculated C–C bond length of the two sides of each phenyl ring attached to ethyl bridges is 1.40 Å compared to its experimental value of 1.390(2) Å while the other four bonds are calculated to be 1.39 Å compared to the experimental 1.387(3) Å bond length.

### *p*Cp–M^*n*+^ and Dp–M^*n*+^ complexes

Inclusion complexes of *p*Cp (*p*Cp–M^*n*+^) and Dp (Dp–M^*n*+^) with nine metal-cations of interest were optimized in their *D*_3_ and *C*_3_ symmetry and confirmed as either minima or transition structures through vibrational analysis. In the former case, the group 13 complexes and *p*Cp–As^3+^ are *C*_3_ symmetric minima while group 14 and the rest of group 15 *p*Cp–M^*n*+^ complexes have *D*_3_ symmetry in their ground state structures. In case of group 13 *p*Cp–M^+^ complexes and *p*Cp–As^3+^, the *D*_3_ symmetry was possible for the transition structures only. The *D*_3_ symmetric *p*Cp–M^*n*+^ complexes show η^6^η^6^η^6^ metal–phenyl rings coordination. However, *p*Cp–Ga^+^ and *p*Cp–As^3+^ present a case with *C*_3_ symmetry showing η^6^η^6^η^6^ coordination while its counterparts *i.e. p*Cp–In^+^ and *p*Cp–Tl^+^ exhibit η^2^η^2^η^2^ coordination. As for the Dp complexes, all the nine structures were optimized as minima in *C*_3_ as well as *D*_3_ symmetry. Dp–M^*n*+^ complexes with *C*_3_ show η^1^η^1^η^1^ coordination of a metal ion staying on the top of Dp cavity in each complex. *D*_3_ symmetric complexes, on the other hand, exhibit η^6^η^6^η^6^ coordination of the metal-cation present inside the Dp cavity. These results suggest that despite few exceptions, the *p*Cp and Dp complexes of main-group metal-cations under discussion differ from the transition metal complexes where the metal-cation tends to come out of the cavity^20,21^ preferring peripheral coordination unlike current complexes where central (η^6^η^6^η^6^) coordination is preferred.

Some key structural parameters of *p*Cp–M^*n*+^ and Dp–M^*n*+^ of interest are given in Table 1. The experimental evidence is available only for *p*Cp–Ga^+^, *p*Cp–In^+^, *p*Cp–Ge^2+^, *p*Cp–Sn^2+^ and *p*Cp–As^3+^ where it can be seen that computational results align well with the experimental ones. However, the distance of metal ion from the center of the *p*Cp cavity in experimental and computational instances differs, which can be attributed to the proximity of corresponding counter anion(s) to the metal cation in experimentally reported complexes. This is in line with the computational findings reported earlier in case of *p*Cp–Sn^2+^.^18^ The difference is highly pronounced in case of *p*Cp–As^3+^ where both the calculated conformations (*C*_3_ and *D*_3_) exhibit η^6^η^6^η^6^ coordination mode, as mentioned earlier, with As^3+^ in *C*_3_ symmetry displaced by 0.266 Å from the center of the host cavity. On the other hand, the experimentally reported *p*Cp–As^3+^ shows η^2^η^2^η^2^ coordination where AsCl_3_ coordinates from the top of the cavity. In addition to *p*Cp–Sn^2+^, the computational findings for *p*Cp–In^+^ have also been reported earlier^22^ which, despite small numerical differences, show a great deal of similarity with the *p*Cp–In^+^ calculated in our case.

**Table:** Key structural features of *p*Cp–M^*n*+^ and Dp–M^*n*+^. Exp. shows experimentally reported *p*Cp–Ga^+^, *p*Cp–In^+^, *p*Cp–Ge^2+^, *p*Cp–Sn^2+^ and *p*Cp–As^3+^. The computational results for *p*Cp–In^+^ and *p*Cp–Sn^2+^ reported earlier given in parentheses. Avg. C–M, Cent–M and C=C–M denote average metal–carbon distances, distance of metal cation from the center of the cavity, and average distance between C=C of phenyl rings (upper and lower in *p*Cp–M^*n*+^ and sideways in Dp–M^*n*+^) from metal ion, respectively

		Avg. C–M	Cent–M	Internal Radius	C=C–M
*p*Cp–Ga^+^	*C*_3_	2.993	0.466	2.603	2.896
	*D*_3_	2.972	0.000	2.625	2.895
	Exp.^17^	2.985	0.417	2.628	2.910
*p*Cp–In^+^	*C*_3_	3.130 (3.170)	1.192 (1.698)	2.644	3.052 (3.091)
	*D*_3_	3.029 (3.101)	0.000 (0.000)	2.689 (2.765)	2.963 (3.042)
*p*Cp–Tl^+^	*C*_3_	3.144	1.351	2.640	3.066
	*D*_3_	3.040	0.000	2.701	2.976
*p*Cp–Ge^2+^	*D*_3_	2.843	0.000	2.476	2.746
	Exp.^18^	3.062	0.994	2.449	3.017
*p*Cp–Sn^2+^	*D*_3_	2.952 (2.975)	0.000 (0.000)	2.599 (2.595)	2.872 (2.877)
	Exp.^18^	2.958	0.382	2.581	2.877
*p*Cp–Pb^2+^	*D*_3_	2.957	0.000	2.605	2.879
*p*Cp–As^3+^	*C*_3_	2.851	0.266	2.498	2.742
	*D*_3_	2.829	0.000	2.418	2.685
	Exp.^19^	3.458	2.799	2.578	3.390
*p*Cp–Sb^3+^	*D*_3_	2.866	0.000	2.498	2.769
*p*Cp–Bi^3+^	*D*_3_	2.900	0.000	2.537	2.810
Dp–Ga^+^	*C*_3_	2.637	2.354	2.365	2.744
	*D*_3_	2.836	0.000	2.467	
Dp–In^+^	*C*_3_	2.863	2.788	2.374	2.779
	*D*_3_	2.877	0.000	2.512	
Dp–Tl^+^	*C*_3_	2.863	2.788	2.374	2.779
	*D*_3_	2.877	0.000	2.512	
Dp–Ge^2+^	*C*_3_	2.910	2.866	2.377	2.792
	*D*_3_	2.891	0.000	2.528	
Dp–Sn^2+^	*C*_3_	2.526	2.219	2.345	2.732
	*D*_3_	2.823	0.000	2.449	
Dp–Pb^2+^	*C*_3_	2.590	2.286	2.360	2.750
	*D*_3_	2.843	0.000	2.472	
Dp–As^3+^	*C*_3_	2.206	2.060	2.230	2.658
	*D*_3_	2.724	0.000	2.343	
Dp–Sb^3+^	*C*_3_	2.378	2.190	2.311	2.670
	*D*_3_	2.784	0.000	2.400	
Dp–Bi^3+^	*C*_3_	2.460	2.253	2.331	2.715
	*D*_3_	2.803	0.000	2.421	

The structural parameters of *C*_3_ and *D*_3_ symmetric conformers of *p*Cp–Ga^+^ are identical except the displacement of Ga^+^ from the center of the cavity by 0.466 Å in *C*_3_ conformer in contrast to its exactly central location in the *D*_3_ counterpart. This is supported by identical Δ*E*^int^ of both the conformers in Table 4. However, in case of *p*Cp–In^+^ and *p*Cp–Tl^+^, the metal ion is located significantly further from the center in *C*_3_ symmetry while it is exactly in the center in *D*_3_ analogue. In both of these complexes, cavity size increases upon inclusion of metal cation for η^6^η^6^η^6^ coordination as evident from internal radius C=C–M (Table 1) while it shrinks when the metal cation approaches from the top of the *p*Cp cavity for η^2^η^2^η^2^ interaction. In line with these observations, *C*_3_ conformers of *p*Cp–In^+^ (Δ*E*^int^ = −51.51 kcal mol^−^) and *p*Cp–Tl^+^ (Δ*E*^int^ = −46.78 kcal mol^−^) are energetically favourable by −4.73 kcal mol^−^ and −5.2 kcal mol^−^ than their *D*_3_ (Δ*E*^int^ = −46.78 kcal mol^−^ for *p*Cp–In^+^ and −41.37 kcal mol^−^ for *p*Cp–Tl^+^) counterparts. Similarly, the *D*_3_ symmetric *p*Cp–As^3+^ is less favourable by 8.47 kcal mol^−^ than its *C*_3_ analogue as evidenced by Δ*E*^int^ (Table 4).

**Table:** Gas-phase enthalpies (kcal mol^−1^) of formation of metal complexes *p*Cp–M^*n*+^ and Dp–M^*n*+^

	*p*Cp	Dp		*p*Cp	Dp		*p*Cp	Dp
Ga^+^	−110.1	−66.7	Ge^2+^	−304.0	−284.1	As^3+^	−683.7	−682.8
In^+^	−95.7	−37.8	Sn^2+^	−259.7	−227.8	Sb^3+^	−566.7	−559.0
Tl^+^	−92.5	−30.3	Pb^2+^	−243.9	−207.5	Bi^3+^	−524.8	−511.7

**Table:** Ionic radii (reported here from literature^40–42^) of metal cations of interest in the current study. All the values are in Å

Ga^+^	0.81 (ref. 40)	Ge^2+^	0.73 (ref. 41)	As^3+^	0.53 (ref. 42)
In^+^	1.04 (ref. 40)	Sn^2+^	0.93 (ref. 41)	Sb^3+^	0.76 (ref. 42)
Tl^+^	1.15 (ref. 40)	Pb^2+^	0.98 (ref. 41)	Bi^3+^	0.93 (ref. 42)

Results of the MZEDA analysis for *p*Cp–M^*n*+^ complexes in *D*_3_ symmetry (results for the possible *C*_3_ symmetric conformers in parentheses) at the PBE0/TZ2P level. Results for *p*Cp–In^+^ and *p*Cp–Sn^2+^ at BP86-D3/TZ2P+ level published earlier^21,22^ given in square brackets for comparison. The percentage shows the contribution of an energy term in the total attraction energy which is the sum of Δ*E*^ele^ and Δ*E*^orb^. All values in kcal mol^−1^

**Table:** 

	Ga		In		Tl	
Δ*E*^prep^	−3.32	(−3.09)	34.55 [10.00]	(−6.80 [4.50])	33.72	(−6.87)
Δ*E*^Pauli^	66.40	(61.73)	89.40 [73.20]	(49.24 [39.10])	88.67	(44.47)
Δ*E*^ele^	−59.47	(−56.98)	−70.74 [−49.10]	(−49.34 [−31.00])	−68.25	(−45.62)
	46.1%	(45.8%)	52.00% [40.80%]	(48.97% [36.90%])	52.50%	(50.10%)
Δ*E*^orb^	−69.46	(−67.40)	−65.44 [−71.20]	(−51.42 [−53.10])	−61.79	(−45.43)
	53.9%	(54.2%)	48.00% [59.20%]	(51.03% [63.10%])	47.5%	(49.9%)
Δ*E*^int^	−62.53	(−62.66)	−46.78 [−58.50]	(−51.51 [−62.00])	−41.37	(−46.57)

**Table:** 

	Ge	Sn	Pb
Δ*E*^prep^	−2.66	−3.77 [2.1]	−3.96
Δ*E*^Pauli^	75.88	88.57 [86.0]	96.88
Δ*E*^ele^	−88.12	−93.57–66.5	−96.56
	26.7%	32.3% [22.4%]	34.2%
Δ*E*^orb^	−242.16	−205.31 [−229.9]	−193.83
	73.3%	68.7% [77.6%]	66.8%
Δ*E*^int^	−254.41	−210.30 [−217.10]	−193.50

**Table:** 

	As		Sb	Bi
Δ*E*^prep^	−10.29	(−7.20)	−4.80	−4.91
Δ*E*^Pauli^	78.98	(130.07)	103.33	106.87
Δ*E*^ele^	−118.37	(−132.27)	−127.73	−129.40
	16.7%	(17.2%)	20.7%	22.4%
Δ*E*^orb^	−589.09	(−634.78)	−488.72	−447.91
	83.3%	(82.8%)	79.3%	77.6%
Δ*E*^int^	−628.51	(−636.98)	−512.72	−470.42

In case of Dp–M^*n*+^, all the *C*_3_ conformers exhibit η^1^η^1^η^1^ coordination with the metal cation located on the top of the cavity where their *D*_3_ analogues are perfectly η^6^η^6^η^6^ coordinated. A comparison of Δ*E*^int^ (Table 5) shows that in most of the cases, *D*_3_ conformers of Dp–M^*n*+^ are energetically favourable compared to their *C*_3_ counterparts except Dp–In^+^, Dp–Tl^+^ and Dp–As^3+^ where η^1^η^1^η^1^ coordination is more favourable.

Results of the MZEDA analysis for Dp–M^*n*+^ complexes in *D*_3_ symmetry (results for the possible *C*_3_ symmetric conformers in parentheses) at the PBE0/TZ2P level. The percentage shows the contribution of an energy term in the total attraction energy which is the sum of Δ*E*^ele^ and Δ*E*^orb^. All values in kcal mol^−1^

**Table:** 

	Ga		In		Tl	
Δ*E*^prep^	−3.76	(−6.20)	−4.81	(−11.67)	−4.78	(−14.01)
Δ*E*^Pauli^	105.14	(72.51)	149.89	(57.30)	149.34	(51.92)
Δ*E*^ele^	−80.18	(−48.98)	−102.52	(−39.82)	−99.57	(−36.36)
	48.4%	(39.3%)	55.4%	(40.3%)	55.9%	(41.3%)
Δ*E*^orb^	−85.48	(−75.66)	−82.92	(−58.98)	−78.54	(−51.59)
	51.6%	(60.7%)	44.6%	(59.7%)	44.1%	(58.7%)
Δ*E*^int^	−60.52	(−52.13)	−35.52	(−41.50)	−28.78	(−36.03)

**Table:** 

	Ge		Sn		Pb	
Δ*E*^prep^	−2.10	(−11.11)	−5.80	(−9.57)	−8.16	(−8.98)
Δ*E*^Pauli^	100.36	(126.50)	140.15	(130.24)	146.76	(120.82)
Δ*E*^ele^	−102.48	(−90.84)	−119.12	(−94.34)	−121.11	(−90.38)
	27.6%	(24.2%)	33.4%	(28.6%)	35.17%	(30.0%)
Δ*E*^orb^	−268.68	(−283.79)	−237.98	(−235.22)	−223.23	(−210.59)
	72.4%	(75.8%)	66.6%	(71.4%)	64.83%	(70.0%)
Δ*E*^int^	−270.81	(−248.13)	−216.92	(−199.32)	−197.60	(−180.15)

**Table:** 

	As		Sb		Bi	
Δ*E*^prep^	−4.48	(−41.84)	−5.16	(−28.21)	−6.11	(−23.29)
Δ*E*^Pauli^	100.30	(201.07)	140.55	(186.07)	154.08	(168.78)
Δ*E*^ele^	−132.95	(−142.09)	−147.66	(−140.83)	−152.61	(−134.77)
	17.4%	(16.2%)	21.6%	(19.7%)	23.5%	(21.8%)
Δ*E*^orb^	−633.37	(−734.81)	−535.01	(−575.48)	−496.03	(−510.29)
	82.6%	(83.8%)	78.4%	(80.3%)	76.5%	(78.2%)
Δ*E*^int^	−666.07	(−675.84)	−542.09	(−530.24)	−494.61	(−476.28)

### Thermodynamic parameters

The trends of thermodynamic stability were established by calculating the enthalpies of reaction for the complexes under discussion given by the reaction as given in eqn (3).

3 Lg + M^*n*+^ → LgM^*n*+^

Here, Lg (ligand) denotes *p*Cp or Dp as the case may be and M^*n*+^ represents the respective metal cation with *n* = 1, 2, 3 for group 13, 14 and 15 respectively. LgM^*n*+^ shows the resulting cyclophane–metal complex.

It can be seen in Table 2 that while moving from left to right in a period, thermodynamic feasibility of *p*Cp or Dp complexes is enhanced as indicated by increasing exothermic enthalpy from group 13 through 14 to 15 in the same period. On the other hand, there is a trend of decreasing thermodynamic feasibility while moving from lighter to heavier elements in a group. These trends can be attributed to the size of ionic radii and formal charges on metal-cations. The smaller the ionic radius (as on the top of a group and the left side of a period), the greater the thermodynamic feasibility of the corresponding complex. Ionic radii of the metal cations under discussion are given in Table 3. Moreover, the role of formal charge has been found crucial in determining the strength of interaction and stability previously in such complexes.^22^ Upon moving from group 13 to 15 in a period, the formal charge in our case increases from +1 to +3 and so does the exothermic enthalpy of reaction. The trends of thermodynamic feasibility can be correlated with the results obtained from EDA and are discussed in the next section.

### Bonding properties

The nature of bonding in the inclusion complexes under discussion was carried out using MZEDA technique, Bader's and NBO analyses. The former decomposes total interaction energy into various energy terms and hence it explains the strength of different interactions. NBO and Bader's analyses predict the type of bonding.

### Morokuma–Ziegler energy decomposition analysis

The results for MZEDA of *p*Cp–M^*n*+^ complexes are given in Table 4. It is to be noted that Δ*E*^orb^ indicates the strength of covalent interaction while Δ*E*^ele^ shows the strength of electrostatic attraction. The ratio of Δ*E*^orb^ to Δ*E*^ele^ explains the relative importance of covalent and ionic interactions *i.e.* the greater the ratio, the higher the percentage of Δ*E*^orb^ will be. The total steric repulsion present in a complex is depicted as Δ*E*^Pauli^. The sum of the above-mentioned three terms accounts for the instantaneous interaction energy Δ*E*^int^. However, it is advised in the literature to describe the overall interaction in terms of three separate quantities; Δ*E*^Pauli^, Δ*E*^ele^ and Δ*E*^orb^.^43^

Table 4 shows that in group 13 *p*Cp–M^+^ complexes, interaction energy is highest on the top of group and lowest in case of *p*Cp–Tl^+^. This is in line with the trends of enthalpy of reaction (Table 2) that depicts a decrease in thermodynamic feasibility down the group. It can also be argued that increasing cationic radius (Table 3) down the group makes the π-donation from cyclophane cavity to metal less convenient. It can be seen that the *C*_3_ symmetric *p*Cp–Ga^+^ has an η^6^η^6^η^6^ coordination with the three aromatic rings of *p*Cp which facilitates an overall stronger interaction. The metal-cation in *p*Cp–In^+^ and *p*Cp–Tl^+^ is located further from the centre of the cavity building an η^2^η^2^η^2^ coordination in each case, thus contributing to a comparatively lower interaction energy.

A deeper insight into the EDA results (Table 4) shows that *D*_3_ symmetric η^6^η^6^η^6^ transition structures of group 13 experiences a greater repulsion than their *C*_3_ symmetric complexes as indicated by Δ*E*^Pauli^. This is then compensated by a greater Δ*E*^prep^ in case of the former compared to the latter. However, both the *C*_3_ and *D*_3_ conformers of *p*Cp–Ga^+^ are η^6^η^6^η^6^ with Ga^+^ locating a little further from the center in *C*_3_ symmetric complex while Δ*E*^int^ is identical for both. Moreover, coordination in *p*Cp–Ga^+^ is facilitated by a higher percentage of orbital interaction (Δ*E*^orb^) than the electrostatic interaction (Δ*E*^ele^). In case of *p*Cp–In^+^ and *p*Cp–Tl^+^, the percentage of Δ*E*^orb^ is less compared to that in *p*Cp–Ga^+^ and Δ*E*^ele^ fraction substantially increases which ultimately accounts for a decrease in strength of coordination down the group as suggested by a decreasing Δ*E*^int^. The strength of both the attraction terms may be expressed as the ratio of Δ*E*^orb^ to Δ*E*^ele^ which is 1.2 for *p*Cp–Ga^+^ while it is 1.0 for each of *p*Cp–In^+^ and *p*Cp–Tl^+^. The trends of EDA results for group 14 and 15 are identical to those of group 13.

It is important to note that Table 4 includes EDA results reported earlier for *p*Cp–In^+^^22^ and *p*Cp–Sn^2+^ (ref. 21) calculated at BP86/TZ2P+ level. Although there is difference between the numerical values of those earlier and current studies which may be attributed to the different methodologies (BP86/TZ2P+ earlier *versus* PBE0/TZ2P current) used in both studies, the trends are identical. For instance, the η^2^η^2^η^2^-coordinated *p*Cp–In^+^ is favoured compared to its η^6^η^6^η^6^ analogue based on their Δ*E*^int^ in both the cases. Similarly, Δ*E*^orb^ is a major attractive term in η^6^η^6^η^6^-coordinated *p*Cp–Sn^2+^ in earlier and current studies as evidenced by the percentages of their Δ*E*^orb^ and Δ*E*^ele^ (Table 4).

The trends of EDA in the case of the Dp complexes under discussion (Table 5) are similar to that for *p*Cp complexes. There occurs a decrease in overall interaction energy from top to bottom in a group. However, Dp–In^+^ and Dp–Tl^+^ exhibit relatively stronger electrostatic interactions compared to their *p*Cp analogues. This is evident from the ratio of Δ*E*^orb^ to Δ*E*^ele^ that is 0.8 for both of these complexes while the same is 1.0 for both of their *p*Cp counterparts. All Dp complexes demonstrate the presence of strong coordination as evident from the interaction energy. However, Table 5 shows that Δ*E*^int^ of *D*_3_ symmetric η^6^η^6^η^6^-coordinated Dp–M^*n*+^ complexes is comparatively higher than that of their *C*_3_ symmetric η^1^η^1^η^1^-coordinated counterparts. This difference can be correlated to the greater contribution of covalent interactions (Δ*E*^orb^) compared to that of electrostatic interactions (Δ*E*^ele^) in overall Δ*E*^int^ in case of *D*_3_ complexes. On the other hand, *C*_3_ symmetric complexes are characterized by higher contribution of Δ*E*^ele^ than that of Δ*E*^orb^ in the overall Δ*E*^int^ which causes a comparatively weaker coordination in *C*_3_ complexes than their *D*_3_ analogues.

It can be deduced from Tables 4 and 5 that on moving from group 13 to 15 in a period, Δ*E*^int^ increases to a great extent from one metal ion to the next (Table 4). This trend can be attributed to the formal charge of a cation as witnessed previously.^22^ In our case, the formal charge is +1, +2 and +3 on group 13, 14, and 15 metal ions whereas Δ*E*^int^ in a period increases in the order of group 13 < group 14 < group 15. Moreover, the percentage of Δ*E*^orb^ successively increases and that of Δ*E*^ele^ subsequently decreases from left to right in a period. This whole discussion suggests that an increase in formal charge of a metal ion strengthens its coordination with the cyclophane host characterized by an increasing Δ*E*^orb^ which further accounts for an increase in overall Δ*E*^int^ along a period.

### QTAIM (Bader's) analysis

**Fig. 2:**
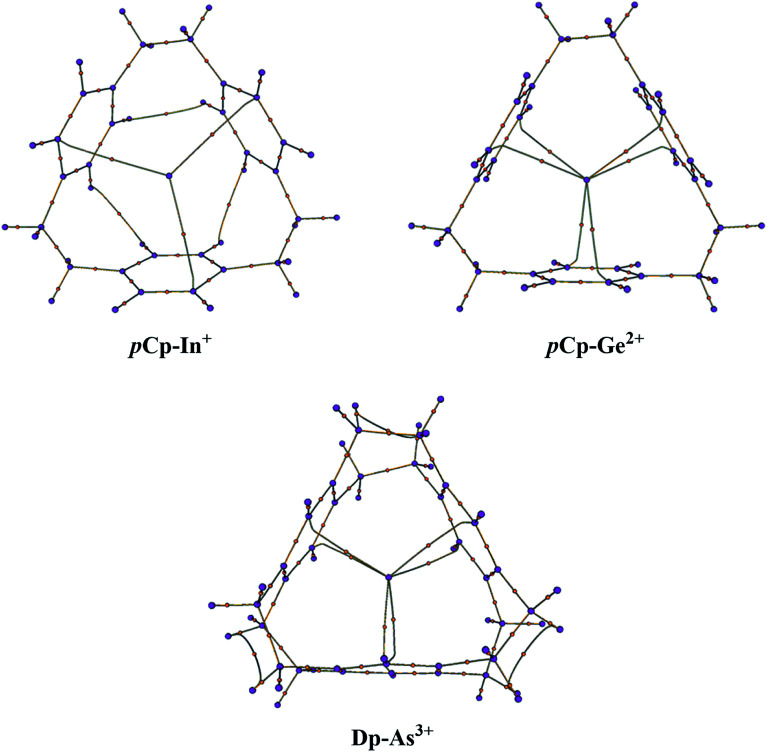
Molecular graphs of *C*_3_ symmetric *p*Cp–In^+^ (representing all the complexes with *C*_3_ symmetry) and *D*_3_ symmetric *p*Cp–Ge^2+^ and Dp–As^3+^ (representing all the *D*_3_ symmetric complexes) calculated at PBE0-B3BJ/def2TZVP. BCPs are shown as orange-coloured dots.

The molecular graphs were extracted from the Bader's analysis results that show the bond paths for all the electron pairs that would be expected for the host molecules *i.e.* CC and CH bonds. Additionally, there are bond critical paths (BCPs) connecting a metal-cation to the host (Fig. 2). For η^2^η^2^η^2^ complexes *i.e. p*Cp–In^+^ and *p*Cp–Tl^+^, there is single BCP between the metal and one carbon atom of each of the aromatic rings toward the surface of the cavity that coordinates with the metal-cation as in the case of *p*Cp–In^+^ in Fig. 2 where the coordination is on the top of the cavity. For all the *p*Cp and Dp *D*_3_ symmetric complexes there are two BCPs connecting the metal from the centre of the cyclophane cavity with two carbon atoms one each on top and bottom sides of the cavity as in the case of *p*Cp–Ge^2+^ and Dp–As^3+^ (Fig. 2). The graphs for *C*_3_ complexes are similar to each other and same is the case with *D*_3_ complexes. This suggests a similar bonding in these complexes.

The results for Bader's analysis of all the complexes are given in Table 6 which may be used to classify the types of interaction in these complexes. For that, Popelier has recently devised a mechanism based on electron density (*ρ*) and its various other functions.^44^ According to the set of these rules, a small *ρ* accompanied by a negative Laplacian L (or ∇^2^*ρ* > 0 since L = −∇^2^*ρ*) usually denotes a depletion of electron density along a BCP and is the characteristic of either closed-shell or donor–acceptor interactions while a small *ρ* along with L approaching zero indicates a shared interaction. On the other hand, a large electron density and a positive L (or ∇^2^*ρ* < 0) shows that electron density is concentrated along a BCP and the interaction will usually be classified as covalent or intermediate. To further confirm the nature of interaction, some additional parameters such as local energy density and the ratio of kinetic energy (*G*) to *ρ* are also advised by Popelier.^44^

**Table:** Electron density (*ρ*) and its Laplacian (L), ratio of kinetic energy to electron density (*G*/*ρ*) and local energy density (*H*) calculated through Bader's analysis of the complexes of interest. These QTAIM parameters may be used as criteria to characterize the type of interaction in the current metal–cyclophane complexes based on Table 8.1 from ref. 44 see discussion in the text. The group 13 *p*Cp–M^+^ and *p*Cp–As^3+^ are *C*_3_ symmetric while all others have *D*_3_ symmetric minima. All values in a.u.

	*p*Cp				Dp			
	*ρ*	L	*G*/*ρ*	*H*	*ρ*	L	*G*/*ρ*	*H*
Ga^+^	0.017	0.036	0.572	−4.0 × 10^−4^	0.024	0.046	0.574	−2.1 × 10^−3^
In^+^	0.016	0.032	0.545	−2.0 × 10^−4^	0.023	0.057	0.634	−1.6 × 10^−3^
Tl^+^	0.018	0.042	0.618	3.0 × 10^−4^	0.027	0.073	0.716	−1.0 × 10^−3^
Ge^2+^	0.025	0.046	0.532	−1.7 × 10^−3^	0.029	0.050	0.540	−2.9 × 10^−3^
Sn^2+^	0.023	0.046	0.565	−1.4 × 10^−3^	0.029	0.058	0.598	−2.8 × 10^−3^
Pb^2+^	0.024	0.059	0.651	−7.0 × 10^−4^	0.030	0.074	0.681	−1.9 × 10^−3^
As^3+^	0.055	0.045	0.428	−1.2 × 10^−2^	0.034	0.056	0.519	−3.6 × 10^−3^
Sb^3+^	0.030	0.050	0.524	−2.8 × 10^−3^	0.034	0.057	0.542	−4.2 × 10^−3^
Bi^3+^	0.028	0.060	0.588	−1.9 × 10^−3^	0.034	0.072	0.625	−3.4 × 10^−3^

A careful classification based on Table 6 suggests that the complexes under discussion possess shared interactions *i.e.* electron sharing is enabled between the cyclophane hosts and the cationic guests. These findings are in line with the results of EDA where Δ*E*^orb^ in most of the cases is a major contributor in total attraction energy compared to Δ*E*^ele^. All these complexes have a small *ρ* and its laplacian (L), a *G*/*ρ* less than 1 and a negative value of *H* except *p*Cp–Tl^+^ that has a positive *H* suggesting that it has interaction between a shared and a donor–acceptor interaction. The EDA results of *p*Cp–Tl^+^ support this assumption since Δ*E*^ele^ has a greater percentage than Δ*E*^orb^.

### Molecular orbital analysis

The electronic structure of the *p*Cp and Dp complexes under consideration was further investigated based on natural population analyses (Table 7). Quantitative molecular orbital (MO) diagrams of *p*Cp–Ga^+^ (*C*_3_ η^6^η^6^η^6^), *p*Cp–In^+^ (*C*_3_ η^2^η^2^η^2^), *p*Cp–Sb^3+^ (*D*_3_ η^6^η^6^η^6^) and Dp–Sn^2+^ (*D*_3_ η^6^η^6^η^6^) are given in Fig. S2 in ESI† to examine bonding and antibonding interactions between the cation and ligand fragments of a complex. The π bonding and antibonding orbitals of a cyclophane ring correspond to those of an aromatic system and have been labelled as π_1_, π_2_ and π_3_. In all the η^6^η^6^η^6^ complexes where the metal cation is centrally located inside the ligand cavity, π_1_ orbital of the cyclophane ligand coordinates with n*s* orbital of metal cation (with *n* = 4, 5, 6 for 4th, 5th and 6th row of elements respectively) giving rise to fully occupied bonding and antibonding π_1_*n*s interactions as in Fig. S2(a)† thus not taking part in overall bonding (‘*n*’ has the value 4, 5, 6 for 4th, 5th, and 6th-row elements respectively). However, *n*p orbital of the metal cation can be bonded to π_2_ and π_3_ orbitals of the aromatic system such that p_*z*_ interacts with π_2_ based on orientation while π_3_ has an equal chance of interaction with p_*x*_ and p_*y*_ orbitals of the metal cation. In *p*Cp–In^+^ and *p*Cp–Tl^+^ complexes, the bonding scheme is somewhat different as both have η^2^η^2^η^2^ coordination mode. We propose in these two cases that only π_2_ and π_3_ of the cyclophane cavity coordinate with *n*s orbital and one of the *n*p sub-orbitals, respectively leaving behind π_1_ without any interaction as in Fig. S2(b).† This is also supported by the comparison of the amount of ligand to metal charge transfer (LMCT) in these two complexes (Table 7) with η^6^η^6^η^6^-coordinated *p*Cp–Ga^+^ where LMCT is double the amount of that in its other two counterparts.

**Table:** NBO data for metal-cations in selected cyclophane–metal complexes. NC shows NBO charges of metal-cations, NEC denotes natural electronic configuration while LMCT is for ligand to metal charge transfer

		*p*Cp	Dp
Ga^+^	NC	0.61	0.57
	NEC	4s^1.98^4p^0.39^	4s^1.98^4p^0.43^
	LMCT	0.39	0.43
In^+^	NC	0.80	0.63
	NEC	5s^1.99^5p^0.19^	5s^1.97^5p^0.37^
	LMCT	0.20	0.37
Tl^+^	NC	0.78	0.64
	NEC	6s^1.98^6p^0.24^	6s^1.98^6p^0.36^
	LMCT	0.22	0.36
Ge^2+^	NC	0.96	0.97
	NEC	4s^1.99^4p^1.03^	4s^1.98^4p^1.02^
	LMCT	1.04	1.03
Sn^2+^	NC	1.16	1.37
	NEC	5s^1.99^5p^0.85^	5s^1.98^5p^0.58^
	LMCT	0.84	0.63
Pb^2+^	NC	1.26	1.40
	NEC	6s^1.99^6p^0.73^	6s^1.98^6p^0.55^
	LMCT	0.74	0.60
As^3+^	NC	0.97	0.92
	NEC	4s^1.99^4p^2.06^	4s^1.98^4p^2.07^
	LMCT	2.03	2.08
Sb^3+^	NC	1.29	1.66
	NEC	5s^1.99^5p^1.71^	5s^1.98^5p^1.28^
	LMCT	1.71	1.34
Bi^3+^	NC	1.37	1.78
	NEC	6s^1.99^6p^1.62^	6s^1.98^6p^1.16^
	LMCT	1.63	1.22

It can be seen in Fig. S2(a)† in MO diagram of *p*Cp–Ga^+^ that π_1_ of *p*Cp and 4s Ga^+^ mix to give fully occupied bonding and antibonding MOs where bonding orbital has 15.87% contribution of Ga^+^ while the remaining 84.13% come from *p*Cp. The contribution of Ga^+^ in antibonding MO, however, increases to 50.90%. Next, π_2_ and 4p_*z*_ mix with a contribution of 6.86% and 83.79% from Ga^+^ in the resultant bonding and antibonding MOs, respectively. Moreover, π_3_ mixes equally with 4p_*x*_ and 4p_*y*_ (as discussed above) to give bonding (1.93% from Ga^+^) and antibonding (60.62% from Ga^+^) interactions. In contrast, we can observe an overall lesser contribution of 5p orbital of In^+^ in Fig. S2(b) compared to 4p of Ga^+^ in (a).† This is in with a higher LMCT in case of η^6^η^6^η^6^-coordinated *p*Cp–Ga^+^ compared to that in η^2^η^2^η^2^-coordinated *p*Cp–In^+^ (Table 7). A comparison of (a), (b), (c) and (d) in Fig. S2† suggests that the greater the contribution of p orbital of corresponding metal cation, the larger the LMCT would be.

### Comparison of *p*Cp and Dp complexes

*p*Cp and Dp have π-rich cavities and can efficiently host the main-group metal-cations making inclusion complexes with them. However, both possess different structures that cause these π-prismands to behave somewhat differently from each other. Although *p*Cp has a rigid geometry as stated earlier, it is still somewhat flexible due to three ethano-bridges linking the three aromatic rings compared to Dp which has six aliphatic chains bridging the three phenyl rings. It readily undergoes conformational changes and tends to adjust its geometry accordingly to accommodate the metal-cations more conveniently. Hence, the *p*Cp complexes are thermodynamically more feasible compared to the Dp complexes.

The comparison is not so simple when it comes to the strength of bonding interaction based on an overall interaction energy. Although, there is no definite trend followed while comparing *p*Cp complexes with their Dp analogues, there are instances where Dp complexes have an increased coordination strength. For example, group 13 *p*Cp–M^+^ complexes (Table 4) have higher interaction energy than corresponding Dp–M^+^ complexes (Table 5) which follows the same trend as in their thermodynamic feasibility (Table 2). However, the interaction energy in case of most of the group 14 and 15 *p*Cp–M^*n*+^ is lower than their Dp analogues. This anomaly can be attributed to the collective effects of Δ*E*^orb^ and Δ*E*^prep^. As a general trend, Δ*E*^orb^ is greater while Δ*E*^prep^ is smaller for Dp–M^*n*+^ complexes compared to *p*Cp–M^*n*+^ complexes.

## Conclusion

Quantum chemical calculations were employed to investigate the pCp and Dp complexes of main-group metals. Geometry optimization under symmetry constraints shows that an η^6^η^6^η^6^ mode of coordination is preferred in most of the cases. These complexes present excellent examples of host–guest interactions unlike the previously reported transition metal complexes of cyclophanes where a metal-cation takes peripheral position on top of the cavity. Most of the complexes are minima in *D*_3_ symmetry whereas group 13 *p*Cp–M^+^ and *p*Cp–As^3+^ have only *C*_3_ symmetric minima. MZEDA shows based on an overall interaction energy that all the complexes of interest bear strong metal–cyclophane coordination. The thermodynamic stability of *p*Cp complexes is higher than their Dp analogues which is as expected due the more flexible and “adjustable” structure of *p*Cp compared to Dp. However, the trends of coordination strength in both the cases are mixed. For example, group 13 *p*Cp complexes have higher interaction energy than their Dp counterparts (a trend that is in line with their thermodynamic stability) while the majority of the rest of Dp complexes have an increased coordination strength compared to the corresponding *p*Cp complexes (thus opposing the trend of thermodynamic stability). This can be correlated with the fact that generally Δ*E*^prep^ is lower and Δ*E*^orb^ is higher for Dp–M^*n*+^ complexes than corresponding *p*Cp–M^*n*+^ except group 13 complexes which result in an overall higher Δ*E*^int^ for Dp–M^*n*+^ than *p*Cp–M^*n*+^ of group 14 and 15. NBO analysis provided the basis for explaining in detail the electronic structure of complexes. Different parameters of Bader's analysis suggest the shared nature of M–C interactions in all these complexes except *p*Cp–Tl^+^ that has a donor–acceptor type of interaction.

## Conflicts of interest

The authors declare that they have no conflict of interest.

## Supplementary Material

RA-010-D0RA05303A-s001
