# Supplementary material for: Main-group metal cyclophane complexes with high coordination numbers
Source: RSC Adv. 2020 Aug 20;10(51):30796–805. doi: 10.1039/d0ra05303a (PMC9056411; doi:10.1039/d0ra05303a)

Electronic Supplementary Material (ESI) for RSC Advances.
This journal is © The Royal Society of Chemistry 2020

S1) Total SCF energy (A.U.) and xyz coordinates of D_3 symmetric complexes (except Group 13 *p*Cp-M^+ and pCp-As^{3+} complexes which have C_3 symmetry), optimized at PBE0-D3BJ/def2TZVP level.

*p*Cp-Ga^+

Energy = -2852.74255678

Atom	X	Y	Z
C	-2.851054	-0.683831	0.096690
C	-2.540826	-0.174113	-1.163965
C	-2.174962	1.156099	-1.331745
C	-2.115866	2.041019	-0.254151
C	-2.515502	1.554476	0.991437
C	-2.869741	0.220249	1.161709
H	-2.548681	-0.827774	-2.028397
H	-1.909111	1.508908	-2.322350
H	-2.520986	2.215865	1.851498
H	-3.136101	-0.134225	2.151949
C	-1.605073	3.443793	-0.437545
H	-1.463212	3.628937	-1.504643
H	-2.365363	4.157674	-0.107996
C	-0.307339	3.766045	0.325409
H	0.000000	4.779270	0.046168

H	-0.514614	3.802291	1.397951
C	0.833312	2.811000	0.096690
C	1.119626	2.287476	-1.163965
C	1.625612	2.375145	1.161709
C	2.088692	1.305523	-1.331745
H	0.557467	2.621110	-2.028397
C	2.603966	1.401251	0.991437
H	1.451808	2.783056	2.151949
C	2.825507	0.811884	-0.254151
H	2.261308	0.898884	-2.322350
H	3.179488	1.075305	1.851498
C	3.784949	-0.331863	-0.437545
H	4.783333	-0.030373	-0.107996
H	3.874358	-0.547290	-1.504643
C	3.415160	-1.616859	0.325409
H	4.138969	-2.389635	0.046168
H	3.550188	-1.455477	1.397951
C	-3.107821	-2.149185	0.325409
H	-3.035574	-2.346814	1.397951
H	-4.138969	-2.389635	0.046168
C	-2.179876	-3.111930	-0.437545
H	-2.417970	-4.127301	-0.107996
H	-2.411146	-3.081647	-1.504643

C	-0.709641	-2.852903	-0.254151
C	-0.088465	-2.955726	0.991437
C	0.086270	-2.461622	-1.331745
C	1.244130	-2.595393	1.161709
H	-0.658502	-3.291170	1.851498
C	1.421199	-2.113363	-1.163965
H	-0.352198	-2.407793	-2.322350
C	2.017742	-2.127169	0.096690
H	1.684293	-2.648831	2.151949
H	1.991214	-1.793336	-2.028397
Ga	0.000000	0.000000	0.405366

----

*p*Cp-In^+

Energy = -1118.29403846

Atom	X	Y	Z
C	0.000000	2.957441	-0.109744
C	0.361981	2.397113	-1.333152
C	1.572949	1.740304	-1.496336
C	2.489003	1.615905	-0.452274
C	2.184529	2.284584	0.735971

C	0.965516	2.939046	0.902451
H	-0.320581	2.446381	-2.171595
H	1.802346	1.299801	-2.459810
H	2.894119	2.272933	1.557190
H	0.746184	3.413907	1.853657
C	3.730035	0.778713	-0.617293
H	3.891887	0.599533	-1.682826
H	4.600975	1.343212	-0.273587
C	3.734623	-0.565122	0.139103
H	4.664028	-1.083799	-0.119674
H	3.794750	-0.372917	1.214384
C	2.561219	-1.478720	-0.109744
C	1.894970	-1.512041	-1.333152
C	2.062530	-2.305684	0.902451
C	0.720673	-2.232366	-1.496336
H	2.278919	-0.945559	-2.171595
C	0.886243	-3.034150	0.735971
H	2.583438	-2.353168	1.853657
C	0.154913	-2.963492	-0.452274
H	0.224488	-2.210778	-2.459810
H	0.521358	-3.642847	1.557190
C	-1.190632	-3.619661	-0.617293
H	-1.137232	-4.656167	-0.273587

H	-1.426733	-3.670239	-1.682826
C	-2.356721	-2.951717	0.139103
H	-3.270612	-3.497267	-0.119674
H	-2.220330	-3.099892	1.214384
C	-1.377901	3.516839	0.139103
H	-1.574420	3.472808	1.214384
H	-1.393416	4.581066	-0.119674
C	-2.539403	2.840948	-0.617293
H	-3.463743	3.312955	-0.273587
H	-2.465154	3.070707	-1.682826
C	-2.643916	1.347587	-0.452274
C	-3.070772	0.749566	0.735971
C	-2.293622	0.492062	-1.496336
C	-3.028046	-0.633362	0.902451
H	-3.415477	1.369915	1.557190
C	-2.256951	-0.885071	-1.333152
H	-2.026834	0.910977	-2.459810
C	-2.561219	-1.478720	-0.109744
H	-3.329622	-1.060739	1.853657
H	-1.958338	-1.500822	-2.171595
In	0.000000	0.000000	0.947137

----

*p*Cp-Tl^{+}

Energy = -1100.67990157

Atom	X	Y	Z
C	-2.918196	-0.443227	-0.283240
C	-2.412028	-0.003140	-1.504043
C	-1.947053	1.292854	-1.668073
C	-1.968448	2.219294	-0.626734
C	-2.588208	1.819828	0.559583
C	-3.051799	0.515671	0.726483
H	-2.355720	-0.686075	-2.341541
H	-1.544406	1.584993	-2.631014
H	-2.692950	2.526621	1.376852
H	-3.499448	0.230469	1.673715
H	-1.328839	3.572408	-0.791789
H	-1.176344	3.759772	-1.857307
H	-2.017102	4.348808	-0.447112
C	0.000000	3.776699	-0.036705
H	0.376876	4.771219	-0.298840
H	-0.198732	3.812498	1.038628
C	1.075253	2.748845	-0.283240
C	1.203294	2.090447	-1.504043

C	1.972483	2.385100	0.726483
C	2.093171	1.039770	-1.668073
H	0.583701	2.383151	-2.341541
C	2.870122	1.331540	0.559583
H	1.949316	2.915376	1.673715
C	2.906189	0.595079	-0.626734
H	2.144847	0.544998	-2.631014
H	3.534594	1.068853	1.376852
C	3.758216	-0.635396	-0.791789
H	4.774729	-0.427542	-0.447112
H	3.844230	-0.861142	-1.857307
C	3.270717	-1.888349	-0.036705
H	3.943558	-2.711994	-0.298840
H	3.401086	-1.734142	1.038628
C	-3.270717	-1.888349	-0.036705
H	-3.202354	-2.078356	1.038628
H	-4.320435	-2.059225	-0.298840
C	-2.429377	-2.937013	-0.791789
H	-2.757627	-3.921265	-0.447112
H	-2.667886	-2.898630	-1.857307
C	-0.937741	-2.814373	-0.626734
C	-0.281913	-3.151368	0.559583
C	-0.146118	-2.332624	-1.668073

C	1.079316	-2.900771	0.726483
H	-0.841643	-3.595474	1.376852
C	1.208733	-2.087307	-1.504043
H	-0.600441	-2.129991	-2.631014
C	1.842944	-2.305619	-0.283240
H	1.550132	-3.145846	1.673715
H	1.772018	-1.697075	-2.341541
Tl	0.000000	0.000000	0.934582

----

*p*Cp-Ge^{2+}

Energy = -3004.50389051

Atom	X	Y	Z
C	-2.857403	0.018335	0.177850
C	-2.566073	0.508057	-1.099192
C	-1.867184	1.702270	-1.271903
C	-1.444580	2.465416	-0.177850
C	-1.723026	1.968256	1.099192
C	-2.407801	0.765894	1.271903
H	-2.891949	-0.040868	-1.975977
H	-1.663141	2.055365	-2.277175

H	-1.410582	2.524935	1.975977
H	-2.611569	0.412640	2.277175
C	-0.673328	3.733544	-0.371627
H	-0.520031	3.901411	-1.439130
H	-1.264743	4.581154	-0.013529
C	0.673328	3.733544	0.371627
H	1.264743	4.581154	0.013529
H	0.520031	3.901411	1.439130
C	1.444580	2.465416	0.177850
C	1.723026	1.968256	-1.099192
C	1.867184	1.702270	1.271903
C	2.407801	0.765894	-1.271903
H	1.410582	2.524935	-1.975977
C	2.566073	0.508057	1.099192
H	1.663141	2.055365	2.277175
C	2.857403	0.018335	-0.177850
H	2.611569	0.412640	-2.277175
H	2.891949	-0.040868	1.975977
C	3.570008	-1.283653	-0.371627
H	4.599767	-1.195278	-0.013529
H	3.638737	-1.500345	-1.439130
C	2.896680	-2.449892	0.371627
H	3.335025	-3.385876	0.013529

H	3.118705	-2.401066	1.439130
C	-3.570008	-1.283653	0.371627
H	-3.638737	-1.500345	1.439130
H	-4.599767	-1.195278	0.013529
C	-2.896680	-2.449892	-0.371627
H	-3.335025	-3.385876	-0.013529
H	-3.118705	-2.401066	-1.439130
C	-1.412823	-2.483751	-0.177850
C	-0.843046	-2.476313	1.099192
C	-0.540617	-2.468164	-1.271903
C	0.540617	-2.468164	1.271903
H	-1.481367	-2.484067	1.975977
C	0.843046	-2.476313	-1.099192
H	-0.948428	-2.468005	-2.277175
C	1.412823	-2.483751	0.177850
H	0.948428	-2.468005	2.277175
H	1.481367	-2.484067	-1.975977
Ge	0.000000	0.000000	0.000000

----

*p*Cp-Sn^{2+}

Energy = -1142.11431938

Atom	X	Y	Z
C	-2.938683	0.056643	0.174466
C	-2.686870	0.578097	-1.101220
C	-1.987293	1.775463	-1.269354
C	-1.518396	2.516653	-0.174466
C	-1.844081	2.037849	1.101220
C	-2.531242	0.833315	1.269354
H	-3.021594	0.039182	-1.981273
H	-1.794609	2.135868	-2.274565
H	-1.544729	2.597186	1.981273
H	-2.747021	0.486243	2.274565
C	-0.672708	3.742972	-0.377411
H	-0.501734	3.878002	-1.447316
H	-1.231546	4.625300	-0.051112
C	0.672708	3.742972	0.377411
H	1.231546	4.625300	0.051112
H	0.501734	3.878002	1.447316
C	1.518396	2.516653	0.174466
C	1.844081	2.037849	-1.101220
C	1.987293	1.775463	1.269354
C	2.531242	0.833315	-1.269354
H	1.544729	2.597186	-1.981273

C	2.686870	0.578097	1.101220
H	1.794609	2.135868	2.274565
C	2.938683	0.056643	-0.174466
H	2.747021	0.486243	-2.274565
H	3.021594	0.039182	1.981273
C	3.577863	-1.288904	-0.377411
H	4.621401	-1.246100	-0.051112
H	3.609315	-1.504487	-1.447316
C	2.905155	-2.454068	0.377411
H	3.389855	-3.379200	0.051112
H	3.107581	-2.373516	1.447316
C	-3.577863	-1.288904	0.377411
H	-3.609315	-1.504487	1.447316
H	-4.621401	-1.246100	0.051112
C	-2.905155	-2.454068	-0.377411
H	-3.389855	-3.379200	-0.051112
H	-3.107581	-2.373516	-1.447316
C	-1.420287	-2.573296	-0.174466
C	-0.842788	-2.615946	1.101220
C	-0.543949	-2.608778	-1.269354
C	0.543949	-2.608778	1.269354
H	-1.476864	-2.636368	1.981273
C	0.842788	-2.615946	-1.101220

H	-0.952412	-2.622111	-2.274565
C	1.420287	-2.573296	0.174466
H	0.952412	-2.622111	2.274565
H	1.476864	-2.636368	-1.981273
Sn	0.000000	0.000000	0.000000

----

*p*Cp-Pb^{2+}

Energy = -1120.69712240

Atom	X	Y	Z
C	-2.942398	0.058837	0.174057
C	-2.692303	0.581828	-1.101312
C	-1.993389	1.779397	-1.269018
C	-1.522154	2.518773	-0.174057
C	-1.850030	2.040689	1.101312
C	-2.537697	0.836628	1.269018
H	-3.029242	0.044270	-1.981497
H	-1.803443	2.141921	-2.274119
H	-1.552960	2.601266	1.981497
H	-2.756680	0.490867	2.274119
C	-0.673105	3.743609	-0.377089

H	-0.502090	3.877708	-1.447154
H	-1.230218	4.627255	-0.051479
C	0.673105	3.743609	0.377089
H	1.230218	4.627255	0.051479
H	0.502090	3.877708	1.447154
C	1.522154	2.518773	0.174057
C	1.850030	2.040689	-1.101312
C	1.993389	1.779397	1.269018
C	2.537697	0.836628	-1.269018
H	1.552960	2.601266	-1.981497
C	2.692303	0.581828	1.101312
H	1.803443	2.141921	2.274119
C	2.942398	0.058837	-0.174057
H	2.756680	0.490867	-2.274119
H	3.029242	0.044270	1.981497
C	3.578613	-1.288879	-0.377089
H	4.622430	-1.248227	-0.051479
H	3.609239	-1.504031	-1.447154
C	2.905508	-2.454731	0.377089
H	3.392211	-3.379028	0.051479
H	3.107148	-2.373677	1.447154
C	-3.578613	-1.288879	0.377089
H	-3.609239	-1.504031	1.447154

H	-4.622430	-1.248227	0.051479
C	-2.905508	-2.454731	-0.377089
H	-3.392211	-3.379028	-0.051479
H	-3.107148	-2.373677	-1.447154
C	-1.420244	-2.577610	-0.174057
C	-0.842274	-2.622517	1.101312
C	-0.544308	-2.616024	-1.269018
C	0.544308	-2.616024	1.269018
H	-1.476282	-2.645535	1.981497
C	0.842274	-2.622517	-1.101312
H	-0.953237	-2.632788	-2.274119
C	1.420244	-2.577610	0.174057
H	0.953237	-2.632788	2.274119
H	1.476282	-2.645535	-1.981497
Pb	0.000000	0.000000	0.000000

----

*p*Cp-As^{3+}

Enerrgy = -3162.78300725

Atom	X	Y	Z
C	-2.767277	-0.755010	0.131000

C	-2.890240	-0.121927	-1.103971
C	-2.522514	1.213501	-1.281212
C	-2.051058	2.002211	-0.226169
C	-1.941655	1.387927	1.029067
C	-2.249075	0.014121	1.199650
H	-3.293834	-0.668967	-1.950303
H	-2.629434	1.658900	-2.265694
H	-1.653371	1.962951	1.901935
H	-2.221860	-0.403155	2.201960
C	-1.642457	3.423445	-0.427491
H	-1.541294	3.623973	-1.495729
H	-2.437494	4.087442	-0.068897
C	-0.343730	3.791354	0.321884
H	0.000000	4.756879	-0.061365
H	-0.535864	3.940157	1.385921
C	0.729781	2.774037	0.131000
C	1.339529	2.563985	-1.103971
C	1.136767	1.940696	1.199650
C	2.312179	1.577811	-1.281212
H	1.067574	3.187027	-1.950303
C	2.172807	0.987559	1.029067
H	0.761788	2.125765	2.201960
C	2.759495	0.775163	-0.226169

H	2.751367	1.447706	-2.265694
H	2.526651	0.450386	1.901935
C	3.786019	-0.289313	-0.427491
H	4.758576	0.067211	-0.068897
H	3.909099	-0.477186	-1.495729
C	3.455274	-1.597998	0.321884
H	4.119578	-2.378439	-0.061365
H	3.680209	-1.506007	1.385921
C	-3.111544	-2.193356	0.321884
H	-3.144344	-2.434151	1.385921
H	-4.119578	-2.378439	-0.061365
C	-2.143562	-3.134132	-0.427491
H	-2.321082	-4.154653	-0.068897
H	-2.367805	-3.146786	-1.495729
C	-0.708437	-2.777374	-0.226169
C	-0.231153	-2.375486	1.029067
C	0.210335	-2.791311	-1.281212
C	1.112309	-1.954817	1.199650
H	-0.873280	-2.413337	1.901935
C	1.550712	-2.442058	-1.103971
H	-0.121933	-3.106607	-2.265694
C	2.037497	-2.019027	0.131000
H	1.460073	-1.722610	2.201960

H	2.226259	-2.518060	-1.950303
As	0.000000	0.000000	0.226874

----

*p*Cp-Sb^{3+}

Energy = -1167.51065110

Atom		X	Y	Z
C	0	-2.878952	0.018540	0.176278
C	0	-2.588024	0.518025	-1.104518
C	0	-1.884918	1.718090	-1.275606
C	0	-1.455532	2.483976	-0.176278
C	0	-1.742635	1.982282	1.104518
C	0	-2.430368	0.773342	1.275606
H	0	-2.921313	-0.022048	-1.985291
H	0	-1.691035	2.075503	-2.282488
H	0	-1.441563	2.540956	1.985291
H	0	-2.642955	0.426728	2.282488
C	0	-0.678522	3.745521	-0.368967
H	0	-0.539547	3.929532	-1.435543
H	0	-1.263427	4.590683	0.009161
C	0	0.678522	3.745521	0.368967

H	0	1.263427	4.590683	-0.009161
H	0	0.539547	3.929532	1.435543
C	0	1.455532	2.483976	0.176278
C	0	1.742635	1.982282	-1.104518
C	0	1.884918	1.718090	1.275606
C	0	2.430368	0.773342	-1.275606
H	0	1.441563	2.540956	-1.985291
C	0	2.588024	0.518025	1.104518
H	0	1.691035	2.075503	2.282488
C	0	2.878952	0.018540	-0.176278
H	0	2.642955	0.426728	-2.282488
H	0	2.921313	-0.022048	1.985291
C	0	3.582977	-1.285143	-0.368967
H	0	4.607362	-1.201182	0.009161
H	0	3.672848	-1.497504	-1.435543
C	0	2.904455	-2.460378	0.368967
H	0	3.343935	-3.389501	-0.009161
H	0	3.133301	-2.432027	1.435543
C	0	-3.582977	-1.285143	0.368967
H	0	-3.672848	-1.497504	1.435543
H	0	-4.607362	-1.201182	-0.009161
C	0	-2.904455	-2.460378	-0.368967
H	0	-3.343935	-3.389501	0.009161

H	0	-3.133301	-2.432027	-1.435543
C	0	-1.423420	-2.502516	-0.176278
C	0	-0.845390	-2.500307	1.104518
C	0	-0.545450	-2.491432	-1.275606
C	0	0.545450	-2.491432	1.275606
H	0	-1.479751	-2.518908	1.985291
C	0	0.845390	-2.500307	-1.104518
H	0	-0.951921	-2.502230	-2.282488
C	0	1.423420	-2.502516	0.176278
H	0	0.951921	-2.502230	2.282488
H	0	1.479751	-2.518908	-1.985291
Sb	0	0.000000	0.000000	0.000000

----

*p*Cp-Bi^{3+}

Energy = -1141.91848668

Atom	X	Y	Z
C	-2.903405	0.029732	0.175073
C	-2.626148	0.540373	-1.105389
C	-1.923465	1.741786	-1.274891
C	-1.477451	2.499556	-0.175073

C	-1.781050	2.004124	1.105389
C	-2.470163	0.794877	1.274891
H	-2.966364	0.005713	-1.987012
H	-1.737782	2.104644	-2.281516
H	-1.488130	2.566090	1.987012
H	-2.691566	0.452641	2.281516
C	-0.678164	3.749958	-0.370458
H	-0.533402	3.923520	-1.438181
H	-1.254463	4.605672	-0.003101
C	0.678164	3.749958	0.370458
H	1.254463	4.605672	0.003101
H	0.533402	3.923520	1.438181
C	1.477451	2.499556	0.175073
C	1.781050	2.004124	-1.105389
C	1.923465	1.741786	1.274891
C	2.470163	0.794877	-1.274891
H	1.488130	2.566090	-1.987012
C	2.626148	0.540373	1.105389
H	1.737782	2.104644	2.281516
C	2.903405	0.029732	-0.175073
H	2.691566	0.452641	-2.281516
H	2.966364	0.005713	1.987012
C	3.586641	-1.287671	-0.370458

H	4.615860	-1.216439	-0.003101
H	3.664569	-1.499820	-1.438181
C	2.908476	-2.462286	0.370458
H	3.361397	-3.389233	0.003101
H	3.131167	-2.423700	1.438181
C	-3.586641	-1.287671	0.370458
H	-3.664569	-1.499820	1.438181
H	-4.615860	-1.216439	0.003101
C	-2.908476	-2.462286	-0.370458
H	-3.361397	-3.389233	-0.003101
H	-3.131167	-2.423700	-1.438181
C	-1.425953	-2.529288	-0.175073
C	-0.845097	-2.544497	1.105389
C	-0.546698	-2.536662	-1.274891
C	0.546698	-2.536662	1.274891
H	-1.478234	-2.571804	1.987012
C	0.845097	-2.544497	-1.105389
H	-0.953784	-2.557285	-2.281516
C	1.425953	-2.529288	0.175073
H	0.953784	-2.557285	2.281516
H	1.478234	-2.571804	-1.987012
Bi	0.000000	0.000000	0.000000

Dp-Ga^+

Energy = -3084.83025296

Atom	X	Y	Z
C	1.276424	2.474759	0.618141
C	1.181264	2.428287	-0.781570
C	-0.092970	2.498414	-1.359776
C	-1.276424	2.474759	-0.618141
C	-1.181264	2.428287	0.781570
C	0.092970	2.498414	1.359776
C	2.693590	-0.191139	0.781570
C	2.781416	-0.131964	-0.618141
C	2.210175	-1.168693	-1.359776
C	1.512326	-2.237148	-0.781570
C	1.504992	-2.342796	0.618141
C	2.117205	-1.329721	1.359776
C	-1.512326	-2.237148	0.781570
C	-1.504992	-2.342796	-0.618141
C	-2.117205	-1.329721	-1.359776
C	-2.693590	-0.191139	-0.781570
C	-2.781416	-0.131964	0.618141

C	-2.210175	-1.168693	1.359776
C	0.757335	-3.454559	1.302816
C	-0.730458	-3.172278	1.670245
C	0.730458	-3.172278	-1.670245
C	-0.757335	-3.454559	-1.302816
C	-3.112502	0.953544	-1.670245
C	-2.613068	2.383151	-1.302816
C	-3.370403	1.071408	1.302816
C	-2.382044	2.218734	1.670245
C	3.370403	1.071408	-1.302816
C	2.382044	2.218734	-1.670245
C	3.112502	0.953544	1.670245
C	2.613068	2.383151	1.302816
H	-0.166220	2.487119	-2.443708
H	0.166220	2.487119	2.443708
H	2.237018	-1.099609	-2.443708
H	2.070798	-1.387510	2.443708
H	-2.070798	-1.387510	-2.443708
H	-2.237018	-1.099609	2.443708
H	0.817794	-4.348461	0.681733
H	1.271830	-3.704912	2.233075
H	-0.761504	-2.758733	2.680180
H	-1.240928	-4.139339	1.730009

H	1.240928	-4.139339	-1.730009
H	0.761504	-2.758733	-2.680180
H	-0.817794	-4.348461	-0.681733
H	-1.271830	-3.704912	-2.233075
H	-4.205237	0.994994	-1.730009
H	-2.769885	0.719885	-2.680180
H	-2.572634	2.953893	-2.233075
H	-3.356981	2.882461	-0.681733
H	-4.174775	1.466001	0.681733
H	-3.844463	0.751020	2.233075
H	-2.964309	3.144345	1.730009
H	-2.008381	2.038848	2.680180
H	4.174775	1.466001	-0.681733
H	3.844463	0.751020	-2.233075
H	2.964309	3.144345	-1.730009
H	2.008381	2.038848	-2.680180
H	4.205237	0.994994	1.730009
H	2.769885	0.719885	2.680180
H	3.356981	2.882461	0.681733
H	2.572634	2.953893	2.233075
Ga	0.000000	0.000000	0.000000

Dp-In^+

Energy = -1350.35778049

Atom	X	Y	Z
C	1.278422	2.515277	0.618877
C	1.182424	2.465358	-0.781593
C	-0.093098	2.556073	-1.359515
C	-1.278422	2.515277	-0.618877
C	-1.182424	2.465358	0.781593
C	0.093098	2.556073	1.359515
C	2.726275	-0.208670	0.781593
C	2.817505	-0.150492	-0.618877
C	2.260173	-1.197411	-1.359515
C	1.543851	-2.256689	-0.781593
C	1.539082	-2.364784	0.618877
C	2.167075	-1.358662	1.359515
C	-1.543851	-2.256689	0.781593
C	-1.539082	-2.364784	-0.618877
C	-2.167075	-1.358662	-1.359515
C	-2.726275	-0.208670	-0.781593
C	-2.817505	-0.150492	0.618877
C	-2.260173	-1.197411	1.359515

C	0.759828	-3.459424	1.301516
C	-0.729157	-3.166488	1.672930
C	0.729157	-3.166488	-1.672930
C	-0.759828	-3.459424	-1.301516
C	-3.106837	0.951776	-1.672930
C	-2.616035	2.387743	-1.301516
C	-3.375863	1.071681	1.301516
C	-2.377681	2.214712	1.672930
C	3.375863	1.071681	-1.301516
C	2.377681	2.214712	-1.672930
C	3.106837	0.951776	1.672930
C	2.616035	2.387743	1.301516
H	-0.166345	2.544666	-2.443561
H	0.166345	2.544666	2.443561
H	2.286918	-1.128274	-2.443561
H	2.120573	-1.416392	2.443561
H	-2.120573	-1.416392	-2.443561
H	-2.286918	-1.128274	2.443561
H	0.809595	-4.355387	0.682549
H	1.264403	-3.718139	2.235132
H	-0.748862	-2.732303	2.674612
H	-1.229275	-4.136842	1.762599
H	1.229275	-4.136842	-1.762599

H	0.748862	-2.732303	-2.674612
H	-0.809595	-4.355387	-0.682549
H	-1.264403	-3.718139	-2.235132
H	-4.197248	1.003838	-1.762599
H	-2.740674	0.717618	-2.674612
H	-2.587801	2.954075	-2.235132
H	-3.367079	2.878823	-0.682549
H	-4.176673	1.476564	0.682549
H	-3.852204	0.764064	2.235132
H	-2.967973	3.133004	1.762599
H	-1.991813	2.014685	2.674612
H	4.176673	1.476564	-0.682549
H	3.852204	0.764064	-2.235132
H	2.967973	3.133004	-1.762599
H	1.991813	2.014685	-2.674612
H	4.197248	1.003838	1.762599
H	2.740674	0.717618	2.674612
H	3.367079	2.878823	0.682549
H	2.587801	2.954075	2.235132
In	0.000000	0.000000	0.000000

Dp-Tl^+

Energy = -1332.73674592

Atom	X	Y	Z
C	1.278201	2.526389	0.620352
C	1.183991	2.482412	-0.781143
C	-0.091320	2.575466	-1.360423
C	-1.278201	2.526389	-0.620352
C	-1.183991	2.482412	0.781143
C	0.091320	2.575466	1.360423
C	2.741827	-0.215840	0.781143
C	2.827017	-0.156240	-0.620352
C	2.276079	-1.208648	-1.360423
C	1.557836	-2.266572	-0.781143
C	1.548816	-2.370149	0.620352
C	2.184759	-1.366818	1.360423
C	-1.557836	-2.266572	0.781143
C	-1.548816	-2.370149	-0.620352
C	-2.184759	-1.366818	-1.360423
C	-2.741827	-0.215840	-0.781143
C	-2.827017	-0.156240	0.620352
C	-2.276079	-1.208648	1.360423
C	0.759789	-3.459826	1.302078

C	-0.733299	-3.171160	1.670350
C	0.733299	-3.171160	-1.670350
C	-0.759789	-3.459826	-1.302078
C	-3.112954	0.950525	-1.670350
C	-2.616403	2.387909	-1.302078
C	-3.376191	1.071917	1.302078
C	-2.379656	2.220635	1.670350
C	3.376191	1.071917	-1.302078
C	2.379656	2.220635	-1.670350
C	3.112954	0.950525	1.670350
C	2.616403	2.387909	1.302078
H	-0.163815	2.567065	-2.444674
H	0.163815	2.567065	2.444674
H	2.305051	-1.141664	-2.444674
H	2.141236	-1.425401	2.444674
H	-2.141236	-1.425401	-2.444674
H	-2.305051	-1.141664	2.444674
H	0.810900	-4.357061	0.684980
H	1.259214	-3.718367	2.238564
H	-0.755539	-2.738092	2.672533
H	-1.225753	-4.145388	1.760493
H	1.225753	-4.145388	-1.760493
H	0.755539	-2.738092	-2.672533

H	-0.810900	-4.357061	-0.684980
H	-1.259214	-3.718367	-2.238564
H	-4.202887	1.011161	-1.760493
H	-2.749027	0.714730	-2.672533
H	-2.590593	2.949695	-2.238564
H	-3.367876	2.880791	-0.684980
H	-4.178776	1.476270	0.684980
H	-3.849807	0.768672	2.238564
H	-2.977135	3.134227	1.760493
H	-1.993488	2.023362	2.672533
H	4.178776	1.476270	-0.684980
H	3.849807	0.768672	-2.238564
H	2.977135	3.134227	-1.760493
H	1.993488	2.023362	-2.672533
H	4.202887	1.011161	1.760493
H	2.749027	0.714730	2.672533
H	3.367876	2.880791	0.684980
H	2.590593	2.949695	2.238564
Tl	0.000000	0.000000	0.000000

Dp-Ge^{2+}

Energy = -3236.62933054

Atom	X	Y	Z
C	1.277920	2.406269	0.620516
C	1.184190	2.361980	-0.783117
C	-0.092757	2.390246	-1.364309
C	-1.277920	2.406269	-0.620516
C	-1.184190	2.361980	0.783117
C	0.092757	2.390246	1.364309
C	2.637630	-0.155451	0.783117
C	2.722850	-0.096423	-0.620516
C	2.116392	-1.114793	-1.364309
C	1.453440	-2.206528	-0.783117
C	1.444930	-2.309846	0.620516
C	2.023635	-1.275453	1.364309
C	-1.453440	-2.206528	0.783117
C	-1.444930	-2.309846	-0.620516
C	-2.023635	-1.275453	-1.364309
C	-2.637630	-0.155451	-0.783117
C	-2.722850	-0.096423	0.620516
C	-2.116392	-1.114793	1.364309
C	0.756902	-3.450090	1.307951
C	-0.734938	-3.187341	1.663255

C	0.734938	-3.187341	-1.663255
C	-0.756902	-3.450090	-1.307951
C	-3.127787	0.957196	-1.663255
C	-2.609414	2.380541	-1.307951
C	-3.366316	1.069549	1.307951
C	-2.392849	2.230145	1.663255
C	3.366316	1.069549	-1.307951
C	2.392849	2.230145	-1.663255
C	3.127787	0.957196	1.663255
C	2.609414	2.380541	1.307951
H	-0.164259	2.382017	-2.448121
H	0.164259	2.382017	2.448121
H	2.145016	-1.048756	-2.448121
H	1.980757	-1.333261	2.448121
H	-1.980757	-1.333261	-2.448121
H	-2.145016	-1.048756	2.448121
H	0.842168	-4.341018	0.686815
H	1.282709	-3.678442	2.236371
H	-0.796700	-2.826687	2.691006
H	-1.262009	-4.146168	1.650764
H	1.262009	-4.146168	-1.650764
H	0.796700	-2.826687	-2.691006
H	-0.842168	-4.341018	-0.686815

H	-1.282709	-3.678442	-2.236371
H	-4.221691	0.980153	-1.650764
H	-2.846332	0.723381	-2.691006
H	-2.544269	2.950080	-2.236371
H	-3.338348	2.899847	-0.686815
H	-4.180515	1.441170	0.686815
H	-3.826979	0.728362	2.236371
H	-2.959683	3.166016	1.650764
H	-2.049633	2.103306	2.691006
H	4.180515	1.441170	-0.686815
H	3.826979	0.728362	-2.236371
H	2.959683	3.166016	-1.650764
H	2.049633	2.103306	-2.691006
H	4.221691	0.980153	1.650764
H	2.846332	0.723381	2.691006
H	3.338348	2.899847	0.686815
H	2.544269	2.950080	2.236371
Ge	0.000000	0.000000	0.000000

Dp-Sn^{2+}

Energy = -1374.22042148

Atom	X	Y	Z
C	1.281099	2.457791	0.622770
C	1.187963	2.414419	-0.782576
C	-0.091286	2.472438	-1.364254
C	-1.281099	2.457791	-0.622770
C	-1.187963	2.414419	0.782576
C	0.091286	2.472438	1.364254
C	2.684930	-0.178403	0.782576
C	2.769059	-0.119431	-0.622770
C	2.186837	-1.157163	-1.364254
C	1.496966	-2.236016	-0.782576
C	1.487960	-2.338360	0.622770
C	2.095551	-1.315275	1.364254
C	-1.496966	-2.236016	0.782576
C	-1.487960	-2.338360	-0.622770
C	-2.095551	-1.315275	-1.364254
C	-2.684930	-0.178403	-0.782576
C	-2.769059	-0.119431	0.622770
C	-2.186837	-1.157163	1.364254
C	0.758259	-3.459627	1.307948
C	-0.736008	-3.189262	1.665970
C	0.736008	-3.189262	-1.665970

C	-0.758259	-3.459627	-1.307948
C	-3.129986	0.957229	-1.665970
C	-2.616995	2.386485	-1.307948
C	-3.375254	1.073142	1.307948
C	-2.393978	2.232033	1.665970
C	3.375254	1.073142	-1.307948
C	2.393978	2.232033	-1.665970
C	3.129986	0.957229	1.665970
C	2.616995	2.386485	1.307948
H	-0.161547	2.473219	-2.448416
H	0.161547	2.473219	2.448416
H	2.222644	-1.096706	-2.448416
H	2.061097	-1.376513	2.448416
H	-2.061097	-1.376513	-2.448416
H	-2.222644	-1.096706	2.448416
H	0.833562	-4.352871	0.688683
H	1.272252	-3.699332	2.240423
H	-0.786504	-2.806841	2.686739
H	-1.249756	-4.155438	1.686549
H	1.249756	-4.155438	-1.686549
H	0.786504	-2.806841	-2.686739
H	-0.833562	-4.352871	-0.688683
H	-1.272252	-3.699332	-2.240423

H	-4.223592	0.995399	-1.686549
H	-2.824048	0.722288	-2.686739
H	-2.567589	2.951469	-2.240423
H	-3.352916	2.898321	-0.688683
H	-4.186478	1.454550	0.688683
H	-3.839841	0.747863	2.240423
H	-2.973837	3.160039	1.686549
H	-2.037544	2.084553	2.686739
H	4.186478	1.454550	-0.688683
H	3.839841	0.747863	-2.240423
H	2.973837	3.160039	-1.686549
H	2.037544	2.084553	-2.686739
H	4.223592	0.995399	1.686549
H	2.824048	0.722288	2.686739
H	3.352916	2.898321	0.688683
H	2.567589	2.951469	2.240423
Sn	0.000000	0.000000	0.000000

Dp-Pb^{2+}

Energy = -1352.79578915

Atom	X	Y	Z

C	1.281347	2.476122	0.624579
C	1.189629	2.435877	-0.781900
C	-0.089578	2.503058	-1.365015
C	-1.281347	2.476122	-0.624579
C	-1.189629	2.435877	0.781900
C	0.089578	2.503058	1.365015
C	2.704346	-0.187689	0.781900
C	2.785058	-0.128381	-0.624579
C	2.212501	-1.173953	-1.365015
C	1.514717	-2.248188	-0.781900
C	1.503710	-2.347740	0.624579
C	2.122923	-1.329105	1.365015
C	-1.514717	-2.248188	0.781900
C	-1.503710	-2.347740	-0.624579
C	-2.122923	-1.329105	-1.365015
C	-2.704346	-0.187689	-0.781900
C	-2.785058	-0.128381	0.624579
C	-2.212501	-1.173953	1.365015
C	0.758974	-3.461906	1.308534
C	-0.738978	-3.193222	1.664046
C	0.738978	-3.193222	-1.664046
C	-0.758974	-3.461906	-1.308534

C	-3.134900	0.956638	-1.664046
C	-2.618611	2.388243	-1.308534
C	-3.377585	1.073662	1.308534
C	-2.395923	2.236584	1.664046
C	3.377585	1.073662	-1.308534
C	2.395923	2.236584	-1.664046
C	3.134900	0.956638	1.664046
C	2.618611	2.388243	1.308534
H	-0.158803	2.509695	-2.449341
H	0.158803	2.509695	2.449341
H	2.252861	-1.117320	-2.449341
H	2.094058	-1.392375	2.449341
H	-2.094058	-1.392375	-2.449341
H	-2.252861	-1.117320	2.449341
H	0.833590	-4.356542	0.691120
H	1.266555	-3.703468	2.244111
H	-0.789640	-2.808338	2.683985
H	-1.244705	-4.163522	1.690090
H	1.244705	-4.163522	-1.690090
H	0.789640	-2.808338	-2.683985
H	-0.833590	-4.356542	-0.691120
H	-1.266555	-3.703468	-2.244111
H	-4.228069	1.003815	-1.690090

H	-2.826912	0.720321	-2.683985
H	-2.574020	2.948603	-2.244111
H	-3.356081	2.900182	-0.691120
H	-4.189672	1.456361	0.691120
H	-3.840575	0.754865	2.244111
H	-2.983364	3.159707	1.690090
H	-2.037273	2.088017	2.683985
H	4.189672	1.456361	-0.691120
H	3.840575	0.754865	-2.244111
H	2.983364	3.159707	-1.690090
H	2.037273	2.088017	-2.683985
H	4.228069	1.003815	1.690090
H	2.826912	0.720321	2.683985
H	3.356081	2.900182	0.691120
H	2.574020	2.948603	2.244111
Pb	0.000000	0.000000	0.000000

Dp-As^{3+}

Energy = -3394.93945427

Atom X Y Z

C	1.281371	2.377411	0.622867
C	1.186547	2.327723	-0.786676
C	-0.094846	2.325017	-1.369128
C	-1.281371	2.377411	-0.622867
C	-1.186547	2.327723	0.786676
C	0.094846	2.325017	1.369128
C	2.609141	-0.136282	0.786676
C	2.699584	-0.079006	-0.622867
C	2.060947	-1.080369	-1.369128
C	1.422594	-2.191441	-0.786676
C	1.418213	-2.298405	0.622867
C	1.966101	-1.244648	1.369128
C	-1.422594	-2.191441	0.786676
C	-1.418213	-2.298405	-0.622867
C	-1.966101	-1.244648	-1.369128
C	-2.609141	-0.136282	-0.786676
C	-2.699584	-0.079006	0.622867
C	-2.060947	-1.080369	1.369128
C	0.763071	-3.447682	1.308901
C	-0.736585	-3.187253	1.660219
C	0.736585	-3.187253	-1.660219
C	-0.763071	-3.447682	-1.308901
C	-3.128534	0.955725	-1.660219

C	-2.604245	2.384680	-1.308901
C	-3.367316	1.063002	1.308901
C	-2.391949	2.231528	1.660219
C	3.367316	1.063002	-1.308901
C	2.391949	2.231528	-1.660219
C	3.128534	0.955725	1.660219
C	2.604245	2.384680	1.308901
H	-0.166160	2.304251	-2.453131
H	0.166160	2.304251	2.453131
H	2.078619	-1.008227	-2.453131
H	1.912460	-1.296024	2.453131
H	-1.912460	-1.296024	-2.453131
H	-2.078619	-1.008227	2.453131
H	0.855657	-4.339891	0.691569
H	1.286450	-3.661843	2.242141
H	-0.813837	-2.859153	2.697092
H	-1.265673	-4.144341	1.605564
H	1.265673	-4.144341	-1.605564
H	0.813837	-2.859153	-2.697092
H	-0.855657	-4.339891	-0.691569
H	-1.286450	-3.661843	-2.242141
H	-4.221941	0.976066	-1.605564
H	-2.883018	0.724773	-2.697092

H	-2.528024	2.945020	-2.242141
H	-3.330628	2.910966	-0.691569
H	-4.186284	1.428925	0.691569
H	-3.814474	0.716823	2.242141
H	-2.956268	3.168275	1.605564
H	-2.069180	2.134380	2.697092
H	4.186284	1.428925	-0.691569
H	3.814474	0.716823	-2.242141
H	2.956268	3.168275	-1.605564
H	2.069180	2.134380	-2.697092
H	4.221941	0.976066	1.605564
H	2.883018	0.724773	2.697092
H	3.330628	2.910966	0.691569
H	2.528024	2.945020	2.242141
As	0.000000	0.000000	0.000000

Dp-Sb^{3+}

Energy = -1399.65530292

Atom	X	Y	Z
C	1.285429	2.420011	0.625701

C	1.192441	2.374936	-0.785101
C	-0.091927	2.405459	-1.369056
C	-1.285429	2.420011	-0.625701
C	-1.192441	2.374936	0.785101
C	0.091927	2.405459	1.369056
C	2.652975	-0.154784	0.785101
C	2.738506	-0.096791	-0.625701
C	2.129152	-1.123119	-1.369056
C	1.460535	-2.220152	-0.785101
C	1.453077	-2.323220	0.625701
C	2.037225	-1.282340	1.369056
C	-1.460535	-2.220152	0.785101
C	-1.453077	-2.323220	-0.625701
C	-2.037225	-1.282340	-1.369056
C	-2.652975	-0.154784	-0.785101
C	-2.738506	-0.096791	0.625701
C	-2.129152	-1.123119	1.369056
C	0.760881	-3.459144	1.311782
C	-0.738980	-3.197603	1.662449
C	0.738980	-3.197603	-1.662449
C	-0.760881	-3.459144	-1.311782
C	-3.138695	0.958826	-1.662449
C	-2.615266	2.388515	-1.311782

C	-3.376148	1.070630	1.311782
C	-2.399715	2.238777	1.662449
C	3.376148	1.070630	-1.311782
C	2.399715	2.238777	-1.662449
C	3.138695	0.958826	1.662449
C	2.615266	2.388515	1.311782
H	-0.161013	2.404605	-2.453688
H	0.161013	2.404605	2.453688
H	2.162956	-1.062861	-2.453688
H	2.001943	-1.341744	2.453688
H	-2.001943	-1.341744	-2.453688
H	-2.162956	-1.062861	2.453688
H	0.850353	-4.352282	0.694810
H	1.277728	-3.683485	2.246410
H	-0.809659	-2.851980	2.694516
H	-1.260374	-4.159987	1.634786
H	1.260374	-4.159987	-1.634786
H	0.809659	-2.851980	-2.694516
H	-0.850353	-4.352282	-0.694810
H	-1.277728	-3.683485	-2.246410
H	-4.232841	0.988477	-1.634786
H	-2.874717	0.724805	-2.694516
H	-2.551127	2.948287	-2.246410

H	-3.344011	2.912568	-0.694810
H	-4.194363	1.439714	0.694810
H	-3.828855	0.735198	2.246410
H	-2.972467	3.171509	1.634786
H	-2.065058	2.127175	2.694516
H	4.194363	1.439714	-0.694810
H	3.828855	0.735198	-2.246410
H	2.972467	3.171509	-1.634786
H	2.065058	2.127175	-2.694516
H	4.232841	0.988477	1.634786
H	2.874717	0.724805	2.694516
H	3.344011	2.912568	0.694810
H	2.551127	2.948287	2.246410
Sb	0.000000	0.000000	0.000000

Dp-Bi^{3+}

Energy = -1374.05481316

Atom	X	Y	Z
C	1.285270	2.434916	0.627450
C	1.193864	2.392649	-0.783783

C	-0.089896	2.434757	-1.369179
C	-1.285270	2.434916	-0.627450
C	-1.193864	2.392649	0.783783
C	0.089896	2.434757	1.369179
C	2.669027	-0.162408	0.783783
C	2.751334	-0.104382	-0.627450
C	2.153509	-1.139526	-1.369179
C	1.475163	-2.230241	-0.783783
C	1.466064	-2.330534	0.627450
C	2.063613	-1.295231	1.369179
C	-1.475163	-2.230241	0.783783
C	-1.466064	-2.330534	-0.627450
C	-2.063613	-1.295231	-1.369179
C	-2.669027	-0.162408	-0.783783
C	-2.751334	-0.104382	0.627450
C	-2.153509	-1.139526	1.369179
C	0.761012	-3.461222	1.312870
C	-0.741534	-3.202220	1.660324
C	0.741534	-3.202220	-1.660324
C	-0.761012	-3.461222	-1.312870
C	-3.143971	0.958922	-1.660324
C	-2.617000	2.389667	-1.312870
C	-3.378012	1.071555	1.312870

C	-2.402436	2.243297	1.660324
C	3.378012	1.071555	-1.312870
C	2.402436	2.243297	-1.660324
C	3.143971	0.958922	1.660324
C	2.617000	2.389667	1.312870
H	-0.157541	2.444049	-2.453911
H	0.157541	2.444049	2.453911
H	2.195379	-1.085590	-2.453911
H	2.037838	-1.358459	2.453911
H	-2.037838	-1.358459	-2.453911
H	-2.195379	-1.085590	2.453911
H	0.851251	-4.355358	0.697322
H	1.273040	-3.687189	2.249778
H	-0.813077	-2.854760	2.691835
H	-1.257053	-4.167814	1.636837
H	1.257053	-4.167814	-1.636837
H	0.813077	-2.854760	-2.691835
H	-0.851251	-4.355358	-0.697322
H	-1.273040	-3.687189	-2.249778
H	-4.237959	0.995267	-1.636837
H	-2.878833	0.723235	-2.691835
H	-2.556679	2.946079	-2.249778
H	-3.346225	2.914884	-0.697322

H	-4.197476	1.440474	0.697322
H	-3.829719	0.741110	2.249778
H	-2.980906	3.172547	1.636837
H	-2.065756	2.131525	2.691835
H	4.197476	1.440474	-0.697322
H	3.829719	0.741110	-2.249778
H	2.980906	3.172547	-1.636837
H	2.065756	2.131525	-2.691835
H	4.237959	0.995267	1.636837
H	2.878833	0.723235	2.691835
H	3.346225	2.914884	0.697322
H	2.556679	2.946079	2.249778
Bi	0.000000	0.000000	0.000000

Figure S2) Molecular orbital (MO) diagrams of few selected cyclophane-metal complexes showing HOMO and LUMO and percentage contribution of orbitals in the resultant MOs.

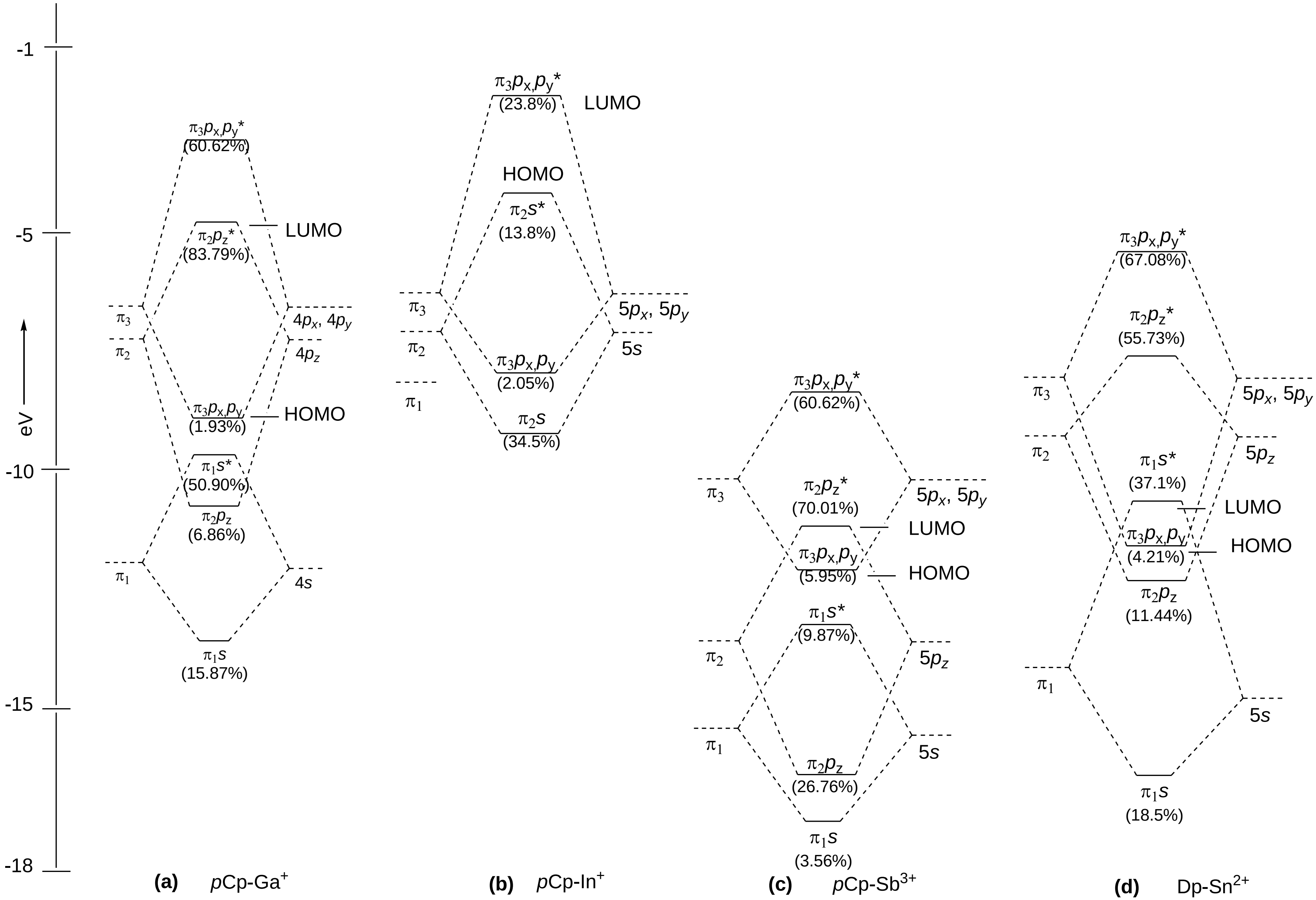

Figure S3) Iso-surfaces of MO interactions of selected *p*Cp-M^{n+} complexes

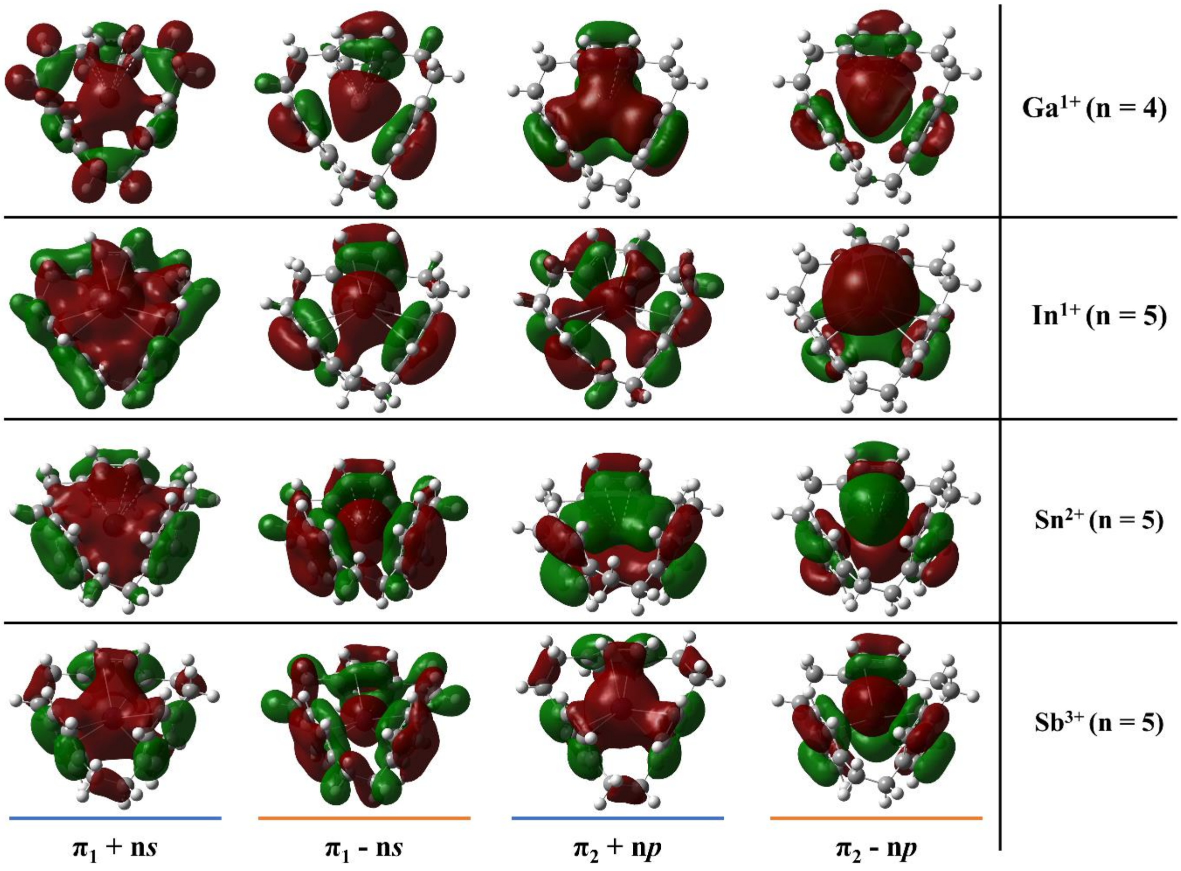

Figure S4) Iso-surfaces of MO interactions of Dp-Sn^{2+}

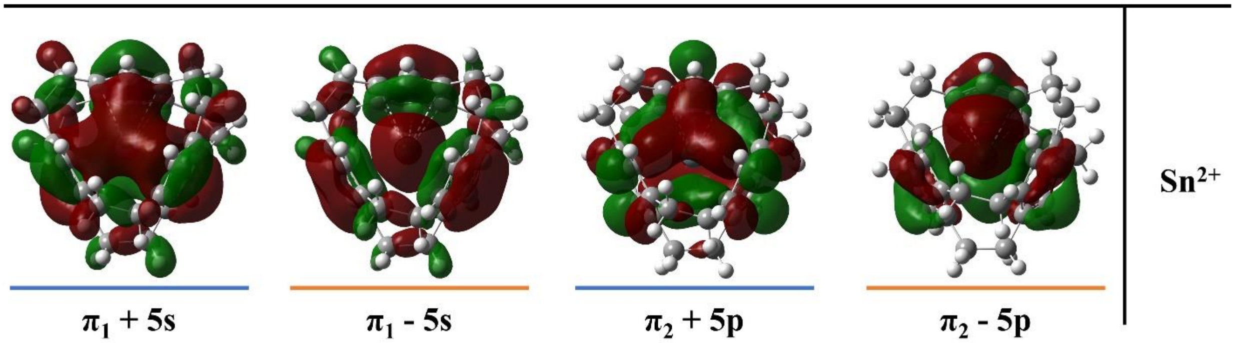

Supplement: RA-010-D0RA05303A-s001 [file RA-010-D0RA05303A-s001.pdf]
